# Encapsulated Mesenchymal Stromal Cells as Cyclic Providers of Immunomodulatory Secretomes: A Living on‐Demand Delivery System

**DOI:** 10.1002/adhm.202304012

**Published:** 2024-04-07

**Authors:** Ana Rita Sousa, Diana C. Gonçalves, Beatriz Guapo Neves, Ana Santos‐Coquillat, Mariana B. Oliveira, João F. Mano

**Affiliations:** ^1^ Department of Chemistry CICECO – Aveiro Institute of Materials University of Aveiro Aveiro 3810‐193 Portugal

**Keywords:** immunomodulation, living materials, on‐demand release systems, stem cells

## Abstract

The stimulation of mesenchymal stromal cells (MSCs) with inflammatory molecules is often used to boost their therapeutic effect. Prolonged exposure to inflammatory molecules has been explored to improve their action because MSCs therapies seem to be improved transiently with such stimuli. However, the possibility of cyclically stimulating MSCs to recover their optimized therapeutic potential is still to be elucidated, although the efficacy of cell‐based therapies may be dependent on the ability to readapt to the relapse pathological conditions. Here, the response of MSCs, encapsulated in alginate hydrogels and cultured for 22 d, is explored using three different regimes: single, continuous, and intermittent stimulation with IFNγ. Exposure to IFNγ leads to a decrease in the secretion of IL‐10, which is cyclically countered by IFNγ weaning. Conditioned media collected at different stages of pulsatile stimulation show an immunomodulatory potential toward macrophages, which directly correlates with IL‐10 concentration in media. To understand whether the correlation between cyclic stimulation of MSCs and other biological actions can be observed, the effect on endothelial cells is studied, showcasing an overall modest influence on tube formation. Overall, the results describe the response of encapsulated MSCs to unusual pulsatile simulation regimens, exploring encapsulated MSCs as a living on‐demand release system of tailored secretomes with recoverable immunomodulatory action.

## Graphical abstract

Secretomes obtained from mesenchymal stem cells encapsulated in alginate hydrogels are modulated for long‐term in vitro culture periods, up to 22 d, using different stimulation regimens with IFNγ. Exposure to IFNγ confers pro‐inflammatory activity to the collected secretomes, and this profile is cyclically recovered with subsequent IFNγ administrations.

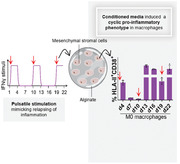

## 1 Introduction

Cell‐derived therapies can be leveraged by understanding how cell responses can be tailored and maximized to meet specific therapeutic demands. Mesenchymal stromal cells (MSCs) are multipotent cells with reported immunomodulatory and regenerative properties. MSCs participate in tissue homeostasis and repair, by secreting stress‐induced anti‐inflammatory and trophic molecules.^[^ ^1^ ^]^ The versatility of MSCs to adapt their response according to microenvironmental cues,^[^ ^2^ ^]^ either contributing to or inhibiting inflammation progression, has been reported.^[^ ^3^ ^]^ Since multiple examples of stem cells in nature experience continuous regenerative cycles,^[^ ^4^, ^5^ ^]^ one could wonder whether MSCs can continuously act in activation/deactivation cycles, according to oscillatory and intermittent environmental stimulus, and whether this response may influence their therapeutic potential. This aspect may be especially relevant to understand how to improve and rescue the therapeutic ability of living cells upon scenarios of, for example, relapsing inflammation, which occur in several inflammatory chronic diseases.^[^ ^6^, ^7^ ^]^ The therapeutic action of MSCs has been ascribed to several cellular components, with the secretome—which includes soluble and insoluble (e.g., extracellular vesicles) factors released to the extracellular space—being the most studied component of MSCs action.^[^ ^8^ ^]^

It is generally accepted that the previous in vitro licensing (also designated as priming) of MSCs with factors found in the inflammatory milieu, including pro‐inflammatory cytokines and/or hypoxia, can improve their therapeutic efficacy toward anti‐inflammatory action and regenerative ability. This effect has been correlated with significant changes in secretome composition.^[^ ^8^ ^]^ So far, however, research on the stimulation of MSCs with pro‐inflammatory molecules has been typically restricted to their short‐term response, with maximum times of analysis of 9 d^[^ ^9^, ^10^, ^11^ ^]^ and with focus on varying doses and duration of the exposure to different stimulatory molecules.^[^ ^12^ ^]^ It has been previously reported, however, that MSCs respond transiently to inflammatory cues, returning to a steady state after stimulus withdrawal.^[^ ^13^, ^14^ ^]^ Some results refer to intracellular molecules and cell surface components with immunomodulatory function, as indoleamine 2,3‐dioxygenase (IDO) and MHC‐II, respectively.^[^ ^13^, ^14^ ^]^ Concerning compositional effects of the secretome, still little is known about the replication of such transient effect. Although biomaterial‐based systems capable of providing prolonged (up to 4 d) exposure of MSCs to pro‐inflammatory molecules have been suggested as effective tools to achieve an also prolonged anti‐inflammatory effect from MSCs,^[^ ^10^ ^]^ the extent of such effect has not been elucidated for long term periods, even after the withdrawal of the stimulus. Understanding the ability of a long‐term stimulation of MSCs to continuously, and possibly consistently, modulate the secretome composition may give insight on the plausible timeframe of effectiveness of these therapies, and how often readministration may be required. Another aspect that remains to be elucidated refers to the ability of MSCs to cyclically recover their ability to be licensed using cycles of stimulus/weaning of pro‐inflammatory molecules. Since continuous exposure to cellular stress may negatively influence stem cell function in the long term,^[^ ^15^ ^]^ determining whether intermittent licensing could help preserve or rescue MSCs’ therapeutic potential could elucidate important cues for the design of future cell therapies.

To address the putative therapeutic potential of the poorly understood effects of stimulus applied in unusual timeframes and cycles to MSCs, we here designed an experimental setup based on the exposure of MSCs to IFNγ, for 22 d, in i) control (unstimulated), ii) one pulse, iii) continuous, and iv) cyclic administration/weaning pulsatile regimens. Human MSCs derived from the adipose tissue (hASCs) were encapsulated in alginate hydrogels, a widely used cell delivery and immobilization platform, enabling the maintenance of cell viability with low proliferation rates. Overall, this study explores a simple system—MSCs encapsulated in alginate hydrogels—as a living on‐demand responsive release device, capable of generating and releasing media with potentially optimized therapeutic efficacy. While administration of MSCs infusions seems safe from a clinical standpoint,^[^ ^16^ ^]^ the ability of these cells to differentiate into different phenotypes, as well as their fast clearance when administered as living suspensions, may contribute to the inconsistent outcomes observed in human trials.^[^ ^16^, ^17^ ^]^ Therefore, such tailored and versatile conditioned media may be used as cell‐free derivative products of MSCs, with more controllable potency, predictability, and stability.^[^ ^18^ ^]^ In addition, understanding such cyclic recovery is expected to pave the way to better understand and explore encapsulated MSCs as predictable living therapeutic agents.

## 2 Results

### 2.1 Exposure to IFNγ Was Mildly Cytotoxic for MSCs Encapsulated in Alginate Matrices for Prolonged Culture Times

**Figure 1:**
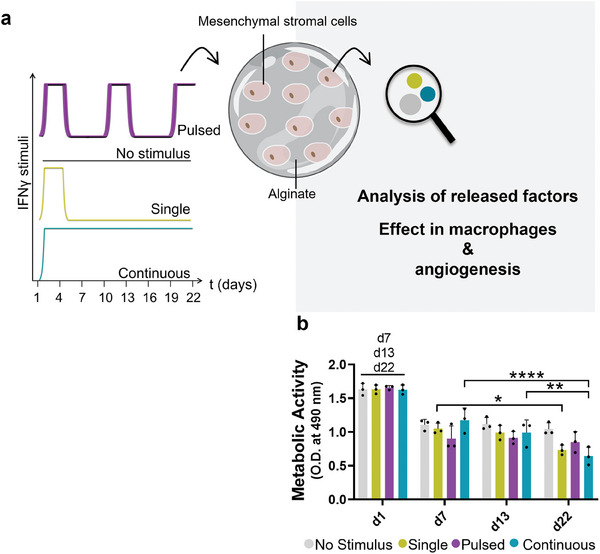
Metabolic activity of mesenchymal stromal cells was minimally reduced after encapsulation into alginate hydrogels during the time of the study. a) Schematic of the study where human mesenchymal stromal cells (MSCs) were encapsulated into alginate hydrogels for 22 d, while exposed to different frequencies of an inflammatory insult consisting in 10 ng mL^−1^ IFNγ for 3 d. The different frequencies consisted of a single (d1–d4), pulsed (d1–d4; d10–d13; d19–d22), and continuous application of the inflammatory stimulus. Every 3 d the medium was changed. A positive control was added, where encapsulated cells received no stimulus. b) Quantification of the metabolic activity of MSCs encapsulated into alginate hydrogels by an MTS test, when submitted to different frequencies of IFNγ stimulus, after 1, 7, 13, and 22 d. As blank control, a condition of empty alginate beads was added. *n* = 3 biological replicates. Error bars are mean ± SD. Statistical differences were determined using two‐way ANOVA followed by Tukey's multiple comparisons test.

We first evaluated if MSCs encapsulation into alginate matrices could be a suitable method to study the role of the frequency of an inflammatory stimulus in the response of MSCs. We hypothesized that, due to the semipermeable properties previously reported for calcium‐crosslinked alginate matrices—enabling the release of secreted factors from cells, and the entrance of low to medium molecular weight molecules from the surrounding environment^[^ ^19^ ^]^—IFNγ would be capable of reaching encapsulated cells, while being washed off from the matrix when needed, enabling the selective administration and weaning of the molecule from the overall system. Importantly, previous data reports show that MSCs keep their immunomodulatory activity while entrapped into alginate matrices.^[^ ^20^ ^]^ hASCs were selected due to their easy isolation,^[^ ^17^ ^]^ as well as to their previously proven response to pro‐inflammatory cytokines as IFNγ.^[^ ^21^ ^]^ The methodology used to stimulate MSCs with IFNγ is depicted in **Figure** **1**a. hASCs were encapsuled in 1.5% (w/v) alginate hydrogels, showcasing an even distribution throughout the whole hydrogels (Figure S1, Supporting Information). Different stimulation regimes were applied (Figure 1a) after 1 d of preculture. A concentration of 10 ng mL^−1^ of IFNγ, within the range of low concentrations used in MSCs stimulation studies,^[^ ^22^, ^23^ ^]^ was used to minimize possible toxicity over time.

In accordance with previous in vitro studies, alginate encapsulation led to a slightly decrease in MSCs viability, approximately after two weeks of culture.^[^ ^20^ ^]^ The addition of IFNγ led to a slight reduction in the metabolic activity and viability of encapsulated cells, in comparison with nonstimulated encapsulated cells (Figures 1b and S2, Supporting Information), mainly for the day 22 timepoint, in which the single and continuous stimulus conditions exhibited a slight reduction in their metabolic activity, with 70.2 ± 3.0% for single and 61.9 ± 15.0% for continuous conditions when compared to the unstimulated control. We further quantified the percentage of live and dead cells area in the fluorescent live/dead images, normalized by the total stained area. Concordantly, some cell death is observed overtime, being the area of dead cells only 20% (from the total stained area) on day 22 (Figure S3, Supporting Information), the last day of the experiment.

### 2.2 Pro‐Inflammatory Stimulation Boosted the Release of Pro‐Regenerative and Immunomodulatory Molecules at Early Time Points

**Figure 2:**
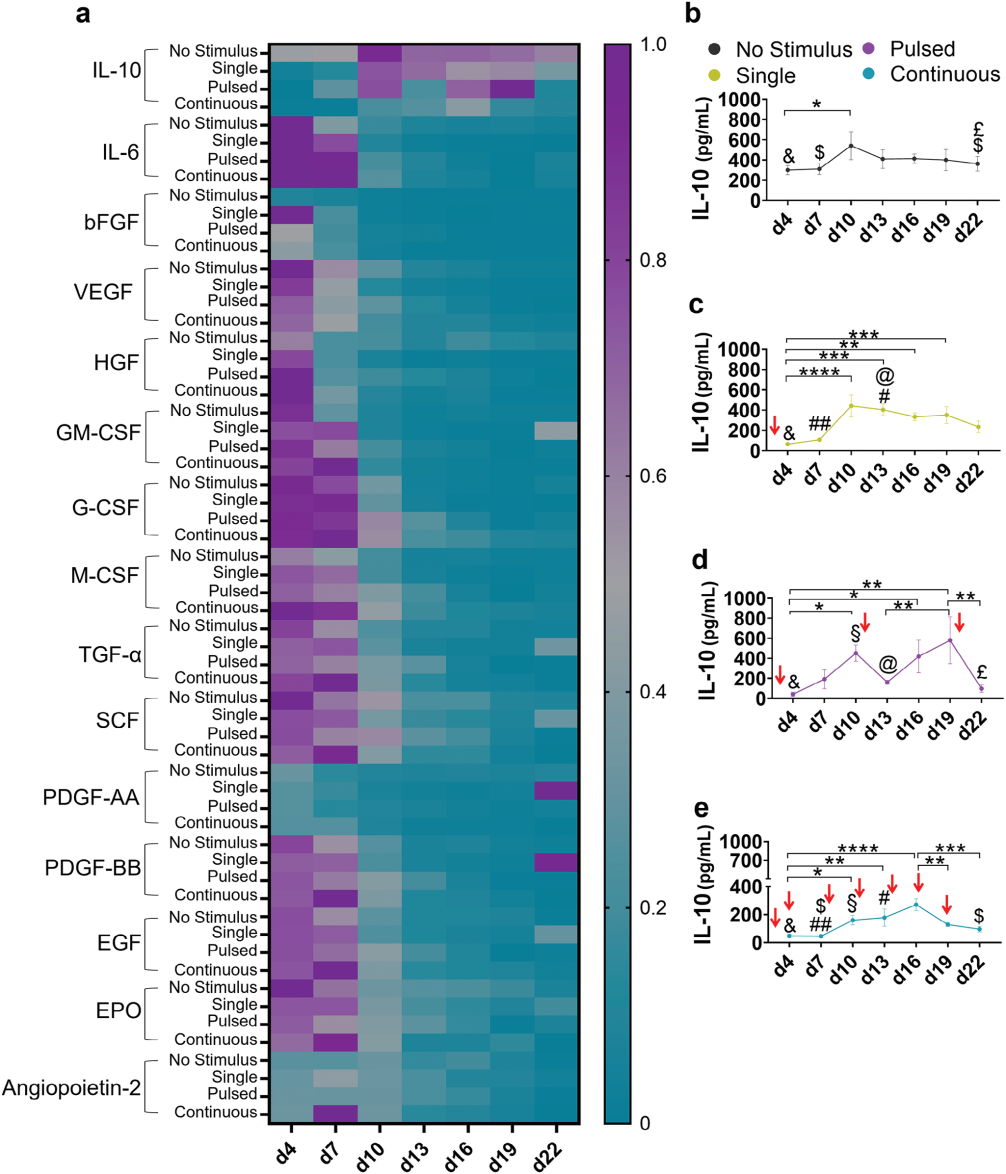
Encapsulated mesenchymal stromal cells cyclically recover a boosted IL‐10 secretion whenever the inflammatory stimulus is withdrawn, over time. a) Heat map of normalized mean levels of multiple growth factors and immunomodulatory molecules released by human mesenchymal stromal cells (MSCs) encapsulated into alginate hydrogels, while exposed to different frequencies of IFNγ stimulus—single, pulsed, and continuous, over 22 d. As a positive control, encapsulated cells receiving no stimulus were added. A multiplex of 13 molecules was analysed by a LEGENDplex kit, while IL‐10 and IL‐6 were analyzed by an ELISA assay. b) Quantification of the secretion of IL‐10 from the encapsulated MSCs, under no inflammatory insult, or c) after the different frequencies of IFNγ stimuli—single, d) pulsed, and e) continuous—, over 22 d. To determine differences over time within each condition, one‐way ANOVA followed by Tukey's multiple comparisons test was used, represented by “*” symbol. To determine differences between different frequencies of inflammatory stimuli, two‐way ANOVA followed by Tukey's multiple comparisons test was used. The symbols “#” refer to significative differences between single and continuous stimulus; “§”: differences between pulsed and continuous stimulus; “&”: differences between positive control and all the frequencies (single, pulsed and continuous stimulus); “$”: differences between positive control and continuous stimulus; “@”: differences between single and pulsed regimens; “£”: differences between positive control and pulsed regimens. Red arrows indicate the timing of IFNγ application. *n* = 3 biological replicates, error bars are mean ± SD.

We evaluated a panel of 15 bioactive interleukins and growth factors known to be secreted by MSCs and previously correlated with immunomodulatory and regenerative actions.^[^ ^1^, ^3^ ^]^ A condition of encapsulated cells receiving no stimulus was included as a control, together with an additional negative control comprising only empty alginate beads. In general, the release of most factors gradually decreased over the course of 22 d of culture (**Figures** **2**a and S4, Supporting Information). IL‐10 was the only factor studied with oscillatory levels over time and will be discussed later. The release of pro‐regenerative factors IL‐6, HGF, as well as the pro‐angiogenic basic FGF (bFGF) was increased following the inflammatory insult until day 4 (for HGF) and day 7 (for both IL‐6 and bFGF), when compared to a positive control which received no stimulus (Figure S4a,b,d, Supporting Information). Accordingly, MSCs are known to promote tissue regeneration and modulation of immune environment upon homing to injured tissues, where MSCs are primed by pro‐inflammatory cytokines as IFNγ or hypoxia, to release many pro‐regenerative factors including HGF, bFGF, and other immunomodulatory molecules.^[^ ^3^ ^]^ Previous in vitro studies showcased that IFNγ‐primed MSCs seeded in 2D cultures increased the secretion of IL‐6^[^ ^9^ ^]^ in comparison with nonstimulated MSCs.^[^ ^24^ ^]^ Discrepant results were found for 3D cultures, where IL‐6 either increased following stimulation of encapsulated MSCs in alginate matrices,^[^ ^20^ ^]^ or decreased when MSCs were primed within protease‐degradable hydrogels,^[^ ^10^ ^]^ or was practically maintained in primed MSCs encapsulated in alginate coupled to an integrin binding peptide.^[^ ^10^ ^]^ The influence of inflammatory stimulation in the response of MSCs has been mostly described for 2D cell culture setups,^[^ ^9^ ^]^ which have reported increase in the secretion of HGF^[^ ^25^ ^]^ and bFGF.^[^ ^3^ ^]^ In our study, the administration of IFNγ also led to an increase in the detected levels of immunosuppressive GM‐CSF, at day 7, and immunomodulatory M‐CSF at days 4 and 7 (Figure S4e,g, Supporting Information). These results are in accordance with previous reports that showed an increase in secreted M‐CSF from MSCs encapsulated in protease‐degradable hydrogels while exposed to IFNγ.^[^ ^10^ ^]^

Overall, the increased release of the factors here reported was observed early after the first administration of IFNγ, and was not replicated after subsequent stimulation cycles (Figure S4a–e,g, Supporting Information). In contrast, the secreted levels of pro‐angiogenic VEGF decreased following IFNγ stimulation, at day 4, but this response was also not reproduced following further inflammatory stimulation (Figure S4c, Supporting Information), with a substantial drop in the secreted values of VEGF after 4 d of culture, for all studied conditions. While some literature supports an increase in the release of VEGF following IFNγ treatment in MSCs cultured as monolayers,^[^ ^3^, ^26^ ^]^ some authors reported a decrease of VEGF secretion in MSCs encapsulated in degradable hydrogels, after 4 d of IFNγ stimulation.^[^ ^10^ ^]^ Previous studies reported the tendency of MSCs to respond transiently to the application of inflammatory stimuli, returning to a steady state after withdrawal.^[^ ^13^, ^14^ ^]^ Here, we corroborated that encapsulated MSCs secreted increased levels of some pro‐regenerative and immunomodulatory factors in response to the first round of inflammatory stimulation (up to day 7); however, this response was gradually lost and was not recovered following subsequent stimulation for the majority of studied factors.

### 2.3 MSCs Encapsulated in Alginate Hydrogels Decreased IL‐10 Secretion after IFNγ Stimulation, Which Was Recovered after IFNγ Withdrawal

Because IL‐10 was the only molecule, from a 15‐molecule panel, responsive to different frequencies of IFNγ stimulation, we further focused on characterizing its secretion in more detail (Figure 2a). A negative control of empty beads was included in the experiment, corroborating that IL‐10 levels in leachables of alginate were lower (mostly undetectable) than the ones released by encapsulated MSCs. The reproducibility of in vitro cell experiments may be affected by multiple variables such as cell culture medium components, namely the different composition of fetal bovine sera from different lots.^[^ ^27^ ^]^ Therefore, we run an additional second independent experiment using medium supplemented with fetal bovine serum (FBS) with a different lot number, as showcased in Figure S5a,b (Supporting Information). An additional third experiment was performed with cells isolated from a different donor. IL‐10 is involved in the modulation of different immune cells, namely in promoting the polarization of macrophages toward an anti‐inflammatory phenotype, relevant to the resolution of inflammation.^[^ ^28^ ^]^ However, it is still controversial whether MSCs increase,^[^ ^29^ ^]^ maintain,^[^ ^11^ ^]^ or decrease^[^ ^30^ ^]^ the secretion of IL‐10 in response to inflammatory stimuli in vitro, mainly due to the lack of available data. This analysis is often unclear, as most studies involve the 2D co‐culture of MSC with other cell types, namely immune cells, remaining uncertain whether the release of IL‐10 in co‐culture supernatants comes from MSCs or from the other cells.^[^ ^3^, ^29^, ^30^ ^]^ In addition, the use of 3D platforms with or without adhesion motifs have shown to modulate the secretion of IL‐10 by hASCs,^[^ ^31^ ^]^ with increased secretion per cell in nonadherent matrices in the case of polyisocyanide hydrogels; also, such compositional differences drove different in vitro regenerative ability of cells. It has also been reported that 3D culture promotes changes in the response of encapsulated MSCs to pro‐inflammatory cytokines, including IL‐1^[^ ^32^ ^]^ although in the latter differences in the anti‐inflammatory potential of secretomes were not identified. Also importantly, the addition of IL‐10 to pro‐inflammatory media was shown as effective in haltering the acquisition of M1 phenotypes in a macrophage cell line in poly(ethylene glycol) (PEG) microgel assembled scaffolds, even in the presence of pro‐inflammatory (LPS/IFNγ) medium.^[^ ^33^ ^]^

Relying on the clear responsivity of IL‐10 secretion from encapsulated MSCs when exposed to IFNγ stimulation, we assessed for how long MSCs can mount a possibly immunomodulatory response, and for how long they can produce a boosted therapeutic effect upon inflammatory stimulation. Encapsulated MSCs receiving no stimulus released close to constant levels of anti‐inflammatory IL‐10 over the 22 d, with a slight increase at day 10 (Figure 2b). However, IFNγ stimulation hindered this stable release, with encapsulated MSCs responding to the inflammatory stimulus with a decrease in the release of IL‐10. Upon a first insult of three days with IFNγ (from day 1 to 4), encapsulated MSCs significantly decreased the release of IL‐10, in comparison with positive control receiving no stimulus, at day 4 (Figure 2c). After that, the IL‐10 levels gradually increased to achieve a steady state from day 10 onwards, reaching similar values to the ones of the control without stimulation. In addition, the IL‐10 levels found from day 10 to day 19 were significantly higher than the ones observed on day 4.

We assessed the ability of encapsulated MSCs to cyclically recover upon IFNγ withdrawn, over time. After each of the three cycles of stimulation, there was a significant decrease in the levels of released IL‐10 (Figure 2d). After the first cycle of stimulus (that lasted from day 1 to day 4), there was a significant increase in IL‐10 levels until day 10, which agrees with the previously described for the single stimulus; in fact, both conditions undergo the same stimulatory pattern until day 10. After a second cycle of IFNγ stimulus (which lasted from day 10 to 13), the levels of detected IL‐10 again decreased, reaching similar (nonstatistically different) levels from the ones found on day 4. Upon the weaning of the stimulus from the system at day 13, encapsulated cells again significantly increased the secreted IL‐10 levels, although to lower values when compared to the recovery after the first cycle. We hypothesize that such lower recovery may be explained by the lower number of viable (and therefore, responsive) cells in the constructs after 13 d of culture (Figures S2 and S3, Supporting Information). Finally, following a third cycle of stimulus (from day 19 to day 22), MSCs significantly decreased the secreted levels of IL‐10. In a second and third independent experiments (different FBS lot and different hASCs donor, respectively), a robust decrease of IL‐10 levels was also seen until the second cycle of stimulus. In the second independent experiment, the IL‐10 decrease seemed to occur to a lower extent in the third cycle of stimulation (Figure S5c, Supporting Information). For the third hASCs experiment, two cycles of IFNγ stimulation effectively led to a decrease in IL‐10 secretion; a third cycle proving the IFNγ‐mediated suppression of IL‐10 secretion could not be performed in this experiment, as it had to be suspended at day 19 (Figure S5e). These results show, for the first time, that alginate‐encapsulated MSCs can cyclically recover an increased IL‐10 secretion when the inflammatory stimulus is withdrawn, provided that a convenient weaning period is applied.

Finally, we evaluated if the persistent exposure of MSCs to an inflammatory stimulus can produce a continuous and stable biological response over time. Concordantly to the observed at day 4 for the single and pulsed conditions, the release of IL‐10 decreased following the first cycle of inflammatory stimulus (Figure 2e). While the IL‐10 release over time remained mostly below the levels found for the control receiving no stimulus, from day 10 to day 16 the IL‐10 levels were significantly higher than the ones observed at day 4. In addition, from day 16 to day 22 there is a significant decrease in IL‐10 secretion. The second independent experiment corroborated the general lower secretion of IL‐10 levels, also exhibiting some significative variations of IL‐10 levels over time (Figure S5d, Supporting Information).

### 2.4 Conditioned Media Retrieved from the Pulsatile Regime Induced a Cyclic Inflammatory Response in Nonpolarized Macrophages

**Figure 3:**
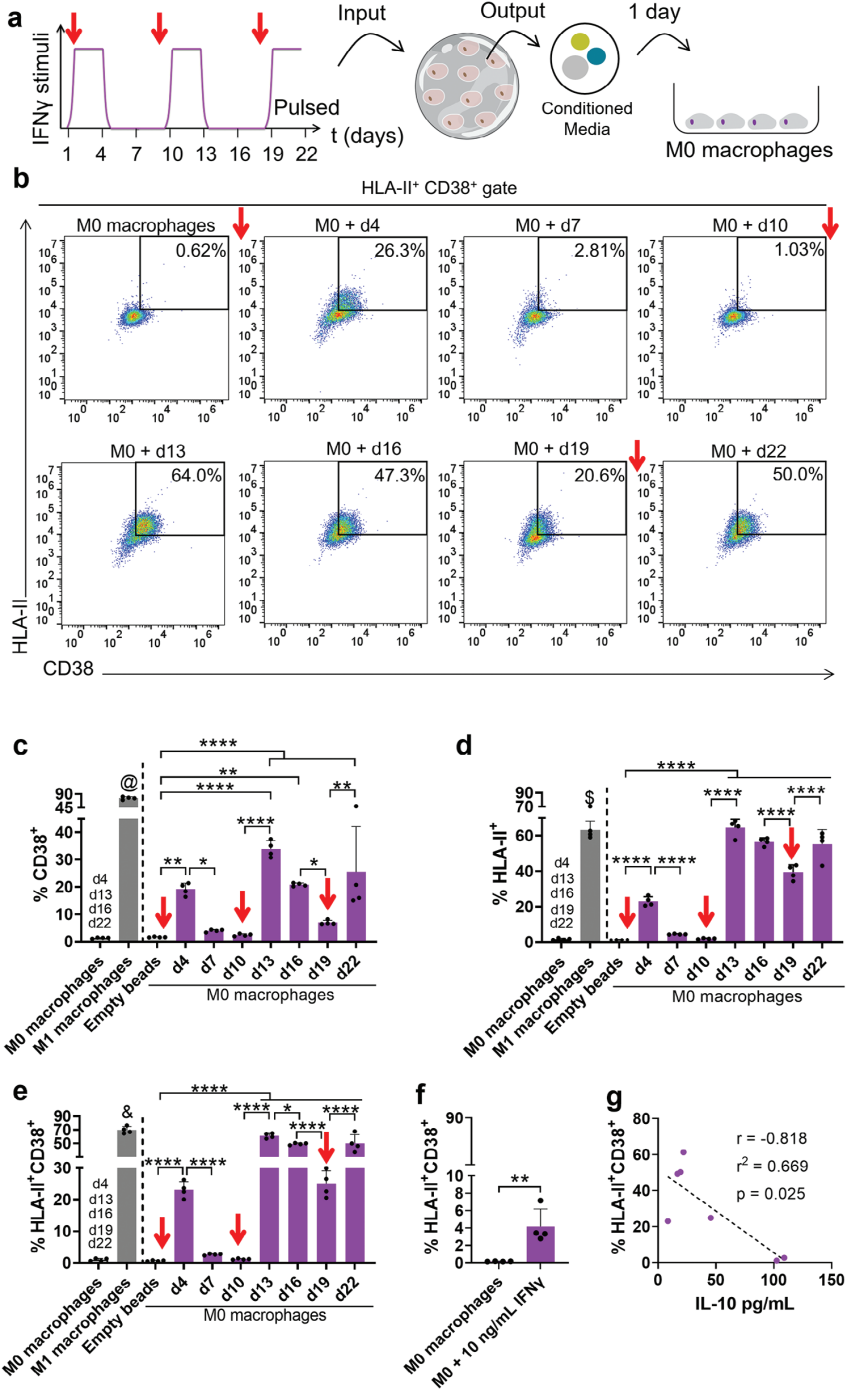
IFNγ stimulus of MSCs leads to secretomes that correlate with pro‐inflammatory phenotype in nonpolarized macrophages. a) Schematic showing the incubation of nonpolarized human THP‐1 macrophages (M0 macrophages) for 1 d with conditioned media from the pulsatile regime collected over the 22 d. b) Representative flow cytometry gating strategy for pro‐inflammatory markers HLA‐II^+^CD38^+^ in nonpolarized macrophages. c) Quantification of the percentage of CD38^+^ cells, d) HLA‐II+ cells and e) HLA‐II^+^CD38^+^ cells. As controls, nonpolarized macrophages were incubated with leachables from empty beads, or with cell culture medium. A positive control for the pro‐inflammatory phenotype included classically polarized macrophages incubated with cell culture medium. Statistical analyses were performed using one‐way ANOVA followed by Tukey's multiple comparisons test. The symbol “*” represents the differences over time within the pulsed condition; on (c), “@” indicates that M1 macrophages were significantly different with *p* < 0.0001 of all remaining conditions; “d4, d13, d16, d22” added over M0 macrophages condition indicates that the nonpolarized macrophages were significantly different with at least *p* < 0.01 from referred days of pulsatile condition. On (d), “$” indicates that M1 macrophages were statistically different with *p* < 0.0001 of all remaining conditions with the exception of conditions of conditioned media collected from the pulsed regime at days d13, d16, and d22, which were nonsignificant; On (e), “&” indicates that M1 macrophages were significantly different with *p* < 0.0001 of all remaining conditions with exception of conditions of media collected from the pulsed regime at d13, that was nonsignificant. On (d) and (e), the “d4, d13, d16, d19, d22” added over M0 macrophages condition indicates that the nonpolarized macrophages were significantly different with *p* < 0.0001 from referred days of pulsatile condition. f) Quantification of the percentage of HLA‐II^+^CD38^+^ cells, while nonpolarized macrophages were incubated for 1 d with cell culture medium or 10 ng mL^−1^ IFNγ supplemented medium. Statistical analyses were performed using unpaired *t*‐test. g) Correlation between the percentage of HLA‐II^+^CD38^+^ cells in a population of nonpolarized macrophages incubated with conditioned media collected from the pulsed regime and the IL‐10 concentration in those media. The correlation was calculated using the parametric Pearson test. All conditioned media, leachables, or cell culture medium used were freeze‐thawed one time before application. *n* = 4 biological replicates, error bars are mean ± SD.

The potential of MSCs conditioned media from the pulsatile regime to differentially induce a pro‐inflammatory response in macrophages was assessed. Macrophages were incubated for 24 h with conditioned media collected over time from the pulsed condition obtained in the second independent experiment (Figure S5c, Supporting Information), which were previously freeze‐thawed once (**Figure** **3**a). Surface markers associated with a pro‐inflammatory phenotype in macrophages (CD38 and HLA‐II)^[^ ^34^, ^35^ ^]^ were analysed. Representative flow cytometry gating strategies for the combined and individual analyses of the markers are depicted in Figures 3b and S6 and S7a,b (Supporting Information). Conditioned media collected after the first cycle of stimulation in encapsulated hMSCs (from day 1 to 4), increased the expression of both CD38 and HLA‐II in nonpolarized macrophages, at day 4, followed by a robust and gradual decrease of pro‐inflammatory markers on days 7 and 10 (Figure 3b–e). As negative controls, nonpolarized macrophages were exposed to either the conditioned medium collected from empty beads condition, or to α‐minimum essential medium (α‐MEM) cell culture medium (M0 macrophages condition), all freeze‐thawed once. The conditioned media from days 7 and 10 did not induce a pro‐inflammatory phenotype in M0 macrophages, supported by the nonsignificant differences found between these conditions and the ones from leachables of empty beads or the M0 macrophages (Figure 3b–e). These results seem coincident with variations of IL‐10 content in pulsed condition (Figures 2d and S5c, Supporting Information), which may be plausible, since IL‐10 is implicated in the metabolic reprogramming of macrophages phenotype.^[^ ^36^ ^]^

Conditioned media collected after the second cycle of stimulation (from day 10 to 13), induced a similar response to the ones collected in the first cycle. The incubation with conditioned media collected at day 13 significantly increased the expression of pro‐inflammatory markers (Figure 3c–e), followed by a decrease until day 19, although to a lower extent than the one observed after the withdrawal of the first cycle of stimulus. The levels of pro‐inflammatory markers upon incubation with conditioned media of day 16 to 19 were significantly higher than the ones observed in negative controls of empty beads and M0 macrophages (Figure 3c–e), with exception for CD38 marker observed upon incubation with conditioned media from day 19, that was nonsignificant (Figure 3c). This oscillatory phenotype after the second cycle seem also to corroborate the respective IL‐10 content of pulsatile media. Upon the second pulsed cycle, higher IL‐10 levels were seen at day 19 (Figures 2d and S5c, Supporting Information), concordant with the largest decrease in pro‐inflammatory markers in nonpolarized macrophages (**Figure** **3c‐e**). Finally, upon the third cycle of stimulation (from days 19 to 22), the media collected at day 22 led to an increase in both pro‐inflammatory markers in nonpolarized macrophages (Figure 3c–e).

**Figure 4:**
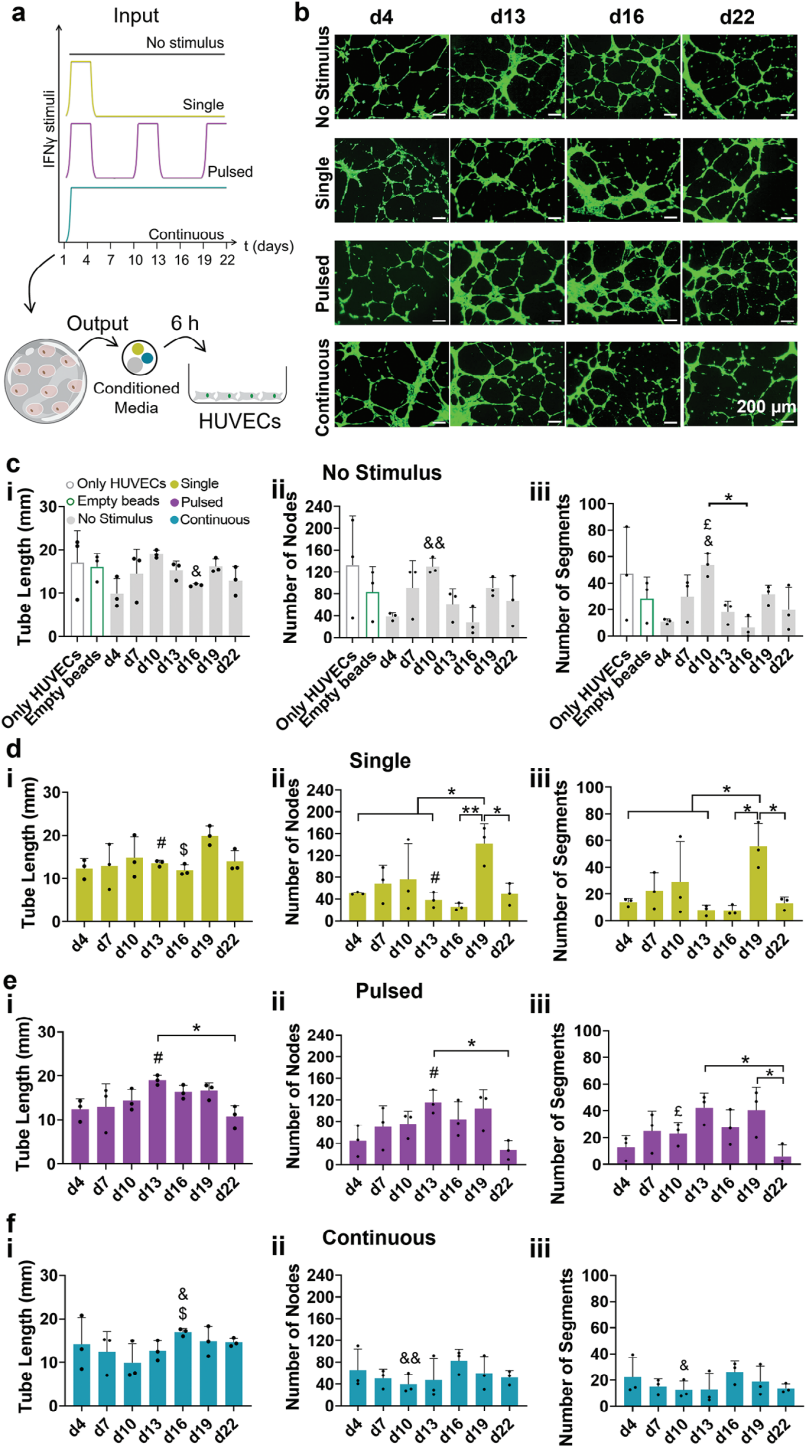
Effect of different conditioned media regimes on angiogenesis. a) Representative fluorescent images of human umbilical vein endothelial cells (HUVECs) stained with calcein‐AM, while incubated for 6 h with a ratio of 1:1 of M199 medium to each conditioned media fashion—single, pulsed, and continuous—and control receiving no stimulus. Scale bar: 200 µm. Quantification of tube length, number of nodes, and number of segments of tube formation for conditions receiving either b) no stimulus, c) single, d) pulsed, and e) continuous stimulus, with IFNγ. As negative controls, HUVECs were either incubated with the leachables of alginate beads (Empty beads) or α‐MEM medium (Only HUVECs). To determine differences over time within each condition of stimuli, one‐way ANOVA followed by Tukey's multiple comparisons test was used, represented by “*” symbol. To determine differences between different frequencies of inflammatory stimuli, within each parameter evaluated, two‐way ANOVA followed by Tukey's multiple comparisons test was used. The symbol “#” refers to significative differences between single and pulsed conditions; “&”: differences between no stimulus and continuous stimulus; “$”: differences between single and continuous stimulus; “£”: differences between no stimulus and pulsed stimulus. *n* = 3 biological replicates, error bars are mean ± SD. All quantifications considered an area of 6 mm^2^.

Overall, the percentage of CD38 positive cells found in conditions incubated with the different conditioned media was significantly lower than a positive control of pro‐inflammatory phenotype incubated in α‐MEM cell culture medium previously freeze‐thawed one time (M1 macrophages, Figure 3c). This indicates that, for the CD38 marker, the response triggered by media collected after inflammatory stimulus on specific days was not as pronounced as a positive control. M0 macrophages incubated with media collected from days 13, 16, and 22 exhibited HLA‐II expression levels nonsignificantly different from the ones observed on M1 macrophages (Figure 3d). Meanwhile, the percentage of CD38^+^HLAII^+^ cells was only not different between M1 macrophages and the media collected from day 13 (Figure 3e).

The IFNγ molecule is widely used to induce the classical polarization of macrophages, toward a pro‐inflammatory M1 phenotype. Since the increase of pro‐inflammatory markers in macrophages is coincident with the timing of IFNγ application in encapsulated MSCs, we run an experiment to understand if the variations in macrophage phenotype may have been caused by possible remnants of IFNγ added to stimulate MSCs. We incubated α‐MEM medium supplemented with IFNγ at 10 ng mL^−1^ for 3 d, followed by freeze‐thawing one time before incubation with macrophages (Figure 3f; Figures S7c and S8, Supporting Information). We observed that the IFNγ supplementation had a neglectable effect in the expression of pro‐inflammatory markers HLA^+^CD38^+^ (4.2 ± 2.0%), in comparison with controls not exposed to IFNγ (0.2 ± 0.0%, Figure 3f). Therefore, the variations observed in the phenotype of macrophages are related to the administered media content, but not to a direct effect of possible remnants of IFNγ added to stimulate encapsulated MSCs.

While IFNγ treatment is used to boost the therapeutic benefits of MSCs secretome,^[^ ^37^ ^]^ it is not clear which are the specific molecules or combinations of secretome components that contribute to phenotypic changes in macrophages.^[^ ^37^, ^38^ ^]^ In our model, there was an inverse correlation between the percentage of macrophages exhibiting pro‐inflammatory markers and the concentration of IL‐10 released by the encapsulated MSCs (Figures 3g and S9, Supporting Information). To better understand whether the presence of higher concentrations of IL‐10 was sufficient to revert a M1 polarized state of macrophages, an additional experiment using M0 macrophages in the presence of conditioned medium collected at day 4 after IFNγ stimulation—proved as pro‐inflammatory—was performed. While a control condition of pulsed CM from day 4 was used after thawing, indeed inducing a pro‐inflammatory phenotype in macrophages (Figure 3b–e), an additional experimental condition comprising pulsatile day 4 CM supplemented with human recombinant IL‐10, at concentrations of 250 pg mL^−1^ (similar to the concentration of IL‐10 detected in CM that did not induce a pro‐inflammatory in macrophages—Figures S10 and S11, Supporting Information) was studied. The effect of the addition of IL‐10 to pro‐inflammatory‐inducing media was previously reported,^[^ ^33^ ^]^ with a concentration of 10 ng mL^−1^ haltering the acquisition of an M1 phenotype by THP‐1 cells. Here, we observed that adding 250 pg mL^−1^ of IL‐10 to the pro‐inflammatory pulsatile day 4 CM was not sufficient to revert its ability to elicit a pro‐inflammatory response in macrophages. Therefore, although IL‐10 content has correlated with the ability of CM to modulate the expression of pro‐inflammatory markers in macrophages, this correlation must be carefully addressed, as the concentration of IL‐10 alone does not show a strict causal relation with the immunomodulatory action of encapsulated MSCs. These results suggest that, along with higher IL‐10 concentration, CM capable of not eliciting inflammatory responses in macrophages probably shows additional compositional changes in their overall compositions, although those could not be detected in the 15‐molecule panel presented in our study.

While other molecules and medium components may participate in the polarization of macrophages, in our model the levels of IL‐10 in the conditioned media seemed useful for a predictive classification of the obtained CM, as pro‐ or non‐inflammatory toward nonpolarized macrophages.

### 2.5 Stimulation of Encapsulated MSCs with IFNγ Generally Decreases Vascular Network Formation In Vitro, and Few Conditions Have a Positive and Modest Impact on Vascular Network Formation

To understand the effect of the different frequencies of IFNγ stimulus on the angiogenic potential of the obtained conditioned media, human primary umbilical vein endothelial cells (HUVECs) were incubated for 6 h in a ratio of 1:1 of M199 medium to conditioned media of all studied conditions (Figure 4a). Representative images of calcein staining (Figure 4b) and representative brightfield images (Figure S12, Supporting Information) showed that independently of the application of IFNγ stimulus, endothelial cells were able to form a network in response to MSCs media collected at early time‐points (from day 4 to 10). However, HUVECs seemed to decrease the quality of the vasculature upon incubation with conditioned media collected at later time points (day 22), exhibiting more cells that did not participate in the network formation. We quantified the tube formation capacity, as well as the number of nodes and segments formed by endothelial cells while exposed to the different media. All conditions generally led to an inferior angiogenic potential than the one observed in the control receiving only α‐MEM medium, previously thawed once to match the same protocol used for conditioned medium storage (Only HUVECs condition, Figure 4c–f). This may be justified since FBS supplement, present in all conditions (including controls) has a proven angiogenic potential in cells.^[^ ^39^ ^]^

All conditions appear to influence angiogenic capacity over time. Overall, variations on the impact on vascular network properties seem sporadic and do not seem to directly correlate with the stimulation of encapsulated MSCs with IFNγ. At day 10, conditioned media from no stimulus control had higher angiogenic potential than pulsed and continuous conditions, namely for the number of segments formed [Figure 4c(iii)]. The same tendency was obtained following calcein staining and for brightfield images (Figure S12, Supporting Information). The control receiving no stimulus reduced the number of segments in endothelial cells from day 10 to 16. Moreover, single stimulus registered an increase in the number of nodes and segments at day 19 [Figure 4d(ii,iii)], but it was not different from all other regimes of IFNγ stimulation. Conditioned media from pulsed condition exhibited higher angiogenic potential than the one seen in single stimulus, at day 13, for both tube length and number of nodes formed [Figure 4e(i,ii)]. This increase in pulsed condition was practically kept from day 13 to 19, followed by a robust decrease at day 22, seen in the number of segments [Figure 4e(iii)]. Calcein staining and the brightfield images support these results and further suggest the improved integrity of the network when endothelial cells are incubated with pulsed media in comparison with the ones of continuous stimulation, from days 13 to 16 (Figures 4b and S12, Supporting Information). However, at day 16, the continuous condition had higher tube length measurements than single and no stimulus conditions [Figure 4f(i)], as indicated by Figure S12. There were no differences in conditioned media collected from continuous stimulus over time [Figure 4f(i–iii)], and the discrepancies observed in calcein staining for continuous and pulsed conditions at day 16 were not transposed to any of the parameters analyzed. Therefore, the conditioned media from encapsulated MSCs exhibited a higher ability to induce the formation of vascular networks in the mid‐term, between days 10 and 16, followed by a generalized decrease on day 22. Overall, IFNγ stimulation seemed not to potently influence the angiogenic potential of MSCs in our system.

## 3 Discussion

Cell‐free derivative products of MSCs such as the secretome have been proposed as a therapeutic option with increased stability to overcome the use of fast‐cleared living MSCs with unpredictable performance following delivery.^[^ ^18^ ^]^ However, there is a lack of understanding and systematization of the mechanisms and parameters influencing the MSCs beneficial properties,^[^ ^8^ ^]^ which compromises the design of engineering strategies to obtain MSCs secretomes with improved therapeutic abilities. The preconditioning of MSCs with inflammatory cues is widely used to boost the therapeutic potential of their secretome,^[^ ^37^ ^]^ but cells return to a steady state after short periods.^[^ ^13^, ^14^ ^]^ Therefore, we addressed here the ability of MSCs to cyclically recover a boosted therapeutic response when the inflammatory stimulus is rather provided intermittently over time. Here, we designed a method to obtain MSCs conditioned media in the long term (22 d) when different frequencies of IFNγ stimulus are applied—single, pulsed, and continuous. MSCs were encapsulated in alginate hydrogels compatible with cell viability, with minimal influence in the long term, and enabled the immunomodulatory activity of MSCs. To the best of our knowledge, we report the unprecedented finding that pulsatile stimulation with IFNγ can induce a cyclic immunomodulatory response in MSCs, over time. Considering the generalized assumption that exposure to IFNγ causes an increase in the anti‐inflammatory profile of MSCs, an unexpected response of alginate‐encapsulated MSCs with a decrease in anti‐inflammatory IL‐10 secretion was observed. This effect was sequentially countered every time the stimulus was weaned from the system. In addition, we observed that the polarization of macrophages to pro‐inflammatory phenotypes directly correlated with the administration of IFNγ to encapsulated cells.

MSCs secretome offer multiple opportunities for therapeutic applications, supported by preclinical and in vitro studies reporting their benefits in, for example, immunomodulation, angiogenesis, fibrosis, and inflammation resolution.^[^ ^37^, ^40^ ^]^ In our model, IFNγ stimulus influenced the secretion of some factors related to pro‐regenerative and immunomodulatory activity, in a transient fashion (up to 6 d upon stimuli removal), and this response was not reset upon subsequent stimulation, except for IL‐10. Moreover, IFNγ stimulation of MSCs influenced the immunomodulatory properties of the obtained conditioned media but seemed not to interfere with its angiogenic potential upon incubation with endothelial cells. Indeed, the priming of MSCs with hypoxia, but not the priming with inflammatory cytokines, was strongly related to the secretion of angiogenic molecules in vitro.^[^ ^9^, ^37^ ^]^ This suggests that controlling cell‐dependent features and manipulation/priming settings might redirect their therapeutic value toward more directed applications. Therefore, understanding the impact of multiple parameters on MSCs outcome, namely of culture conditions—2D or 3D with or without different supporting matrices^[^ ^2^, ^41^ ^]^—, cell preparation features such as the donor or retrieval location,^[^ ^17^ ^]^ the type of stimuli applied,^[^ ^9^ ^]^ and the way it is applied—dose, duration,^[^ ^12^ ^]^ and the here reported frequency—, could help predict differences in the therapeutic potential of MSCs‐derived secretomes. Our results also pave the way for using encapsulated MSCs to design advanced secretome tailoring‐and‐release systems with on‐demand compositions. In addition, by proving that MSCs encapsulated in alginate hydrogels release secretomes capable of inducing a pro‐inflammatory phenotype in nonpolarized macrophages, this gives insight about the possible need for the preparative treatment of defected tissues with additional anti‐inflammatory drugs, in order to achieve the most effective action from MSCs administered in this setting. In addition, our results also call for the need to monitor the local inflammation status of the MSCs‐treated sites, as recurring outbursts of inflammatory response may have negative consequences in the performance of MSCs as pro‐regenerative units, and give insights about the possible exploration of these secretomes as anti‐angiogenic therapeutics.

## 4 Conclusion

We here show a system that enables the cyclical stimulation of MSCs, with the subsequent obtention of conditioned media with immunomodulatory properties. Molecular cocktails with different immunomodulatory and regenerative abilities, and their subsequential on‐demand delivery, may find application in the treatment of diseases and conditions that require different resolution phases, namely concerning the tailoring of different inflammatory states. This model may also find application in the prediction of the performance of encapsulated MSCs after implantation, in different inflammation scenarios comprising recurrency of inflammation, and provide cues for mechanisms happening in stem cell niche following injury.

## 5 Experimental Section

### Cell Culture

hASCs, human THP‐1 cell line, and HUVECs were purchased from ATCC (cat.: PCS‐500‐011, TIB‐202, and PCS‐100‐010, respectively) and Lonza (PT‐5006, Lot 22TL10499). All cells were cultured at 37 °C, in humidified conditions at 5% CO_2_. Human ASCs were cultured with α‐MEM (Gibco), supplemented with 2.2 g L^−1^ sodium bicarbonate (Sigma‐Aldrich), 10% (V/V) FBS (Gibco), and 1% (V/V) antibiotic/antimycotic solution (ATB, Gibco, 10 000 U mL^−1^ of penicillin, 10 000 µg mL^−1^ of streptomycin, and 25 µg mL^−1^ of Amphotericin B) at pH 7.4. Upon confluency of ≈80%, cells were passaged by detaching with trypsin‐EDTA solution (ThermoFisher Scientific) and washed using phosphate buffered saline, without Ca^2+^ and Mg^2+^ (DPBS, Bioconcept). Human ASCs with passage number less than 8 were used for this study. Human THP‐1 cells were cultured in RPMI 1640 (ThermoFisher Scientific), supplemented with 1.2 g L^−1^ sodium bicarbonate, 10 × 10^−3^ m HEPES (Sigma), 1 × 10^−3^ m sodium pyruvate (ThermoFisher Scientific), 10% (V/V) fetal bovine serum, and 1% (V/V) antibiotic/antimycotic solution. HUVEC cells were cultured in M199 medium (Sigma‐Aldrich), supplemented with 2.2 g L^−1^ sodium bicarbonate, 20% (V/V) fetal bovine serum, 1% (V/V) antibiotic/antimycotic solution, 1% (V/V) glutaMAX (ThermoFisher Scientific), 0.1% (W/V) heparin (PanReac), and 0.01% (V/V) of endothelial cell growth supplement (ECGS, Corning).

### Cell Encapsulation and Inflammatory Stimulus

Alginate solution was made with 1.5% (W/V) of alginic acid sodium salt, high viscosity, Mw 10–600 kDa (cat.: A3249, PanReac) in DPBS. Afterward, the alginate solution was sterilized through a 0.22 µm filter (Sigma Aldrich). Human ASCs were then encapsulated at a concentration of 5 × 10^6^ cells mL^−1^ in 1.5% alginate hydrogels, and 20 µL of the cell‐laden hydrogel was added to polydimethylsiloxane (Sylgard 184, Dow Corning) molds, containing cylindrical defects of 5 mm in diameter and 3 mm height. Then, 40 µL of a sterile solution of 0.1 m calcium chloride (cat.: C5670, Sigma‐Aldrich) dissolved in distilled water was added on top of the gels and left reticulating for 30 min, RT. Six cell‐laden hydrogels were added to each well of a 24‐well plate of suspension culture and washed one time with DPBS to remove nonencapsulated cells and incubated with 900 µL α‐MEM complete medium, 37 °C in an incubator. On the day after (day 1), the medium was removed and multiple frequencies of IFNγ stimulus were applied for 22 d, and the conditioned media was collected every 3 d and frozen at −80 °C. For the positive control, the medium was simply collected and changed every 3 d. For the single stimulus, the medium was only supplemented with 10 ng mL^−1^ of recombinant human IFNγ (cat.: 570206, Biolegend) from day 1 to 4. For the pulsed stimulus, the medium was supplemented with 10 ng mL^−1^ of IFNγ and left incubating for 3 d, performing three cycles of stimulation, from day 1 to 4, from day 10 to 13, and from day 19 to 22. In the continuous stimulus, the medium was changed every 3 d and always supplemented with 10 ng mL^−1^ IFNγ. Before adding fresh medium, the hydrogels were washed three times, 1 h each, with 900 µL α‐MEM complete medium, at 37 °C in an incubator, to guarantee that each time the medium is changed (every 3 d) there is no contribution of remaining IFNγ present in the wells. Wells containing only empty alginate hydrogels were used as controls.

### Conditioned Media Analyses

The cytokines IL‐6 and IL‐10 were quantified by ELISA kits according to supplier instructions, using ELISA MAX Standard Set Human IL‐6 (cat.: 430501, BioLegend) and ELISA MAX Standard Set Human IL‐10 (cat.: 430601, BioLegend), respectively. To assess the conditioned media content in multiple pro‐regenerative molecules and trophic factors, it was used a LEGENDplex Human Growth Factor Panel (13‐plex, cat.: 740180, BioLegend) comprising the quantification of 13 human growth factors: Angiopoietin‐2, EGF, EPO, basic FGF, G‐CSF, GM‐CSF, HGF, M‐CSF, PDGF‐AA, PDGF‐BB, SCF, TGF‐α, and VEGF. Samples were prepared as recommended by the manufacturer and data was acquired in a BD Accuri C6 Plus flow cytometer (BD Biosciences). Data were analyzed with LEGENDplex software. Samples from empty beads condition were analyzed as a negative control to confirm that the leachables released from alginate hydrogels in a serum‐supplemented medium contained residual and neglectable levels of the molecules studied.

### Viability and Cytotoxicity Assays

To access the viability of the encapsulated cells, the cell‐laden hydrogels were incubated for 30 min, 37 °C in an incubator, with calcein‐AM (ThermoFisher Scientific) and propidium iodide (PI; ThermoFisher Scientific), at 1:250 and 1:500, respectively, in DPBS. Afterward, the hydrogels were washed once with DPBS and their viability was observed by widefield fluorescence microscopy (AxioImager2, Zeiss). In addition, the metabolic activity of the cell‐loaded hydrogels was accessed by CellTiter 96 AQueous One Solution Cell Proliferation Assay (MTS, Promega), by adding 20% (V/V) of MTS reagent to the wells containing cells in α‐MEM, followed by incubation for 8 h at 37 °C in an incubator. Wells containing only empty beads incubated in the MTS reagent mixture were used as controls. The optical density at 490 nm was measured using Synergy HTX microplate reader. Quantification of the area of living and dead cells was performed based on the analysis of the live/dead fluorescence images, using the Analyze Particles function in ImageJ software.

### Classical Polarization of Monocyte‐Derived Macrophages

Macrophages derived from THP‐1 monocytic cell line (M0 phenotype) were obtained after seeding of 172 000 THP‐1 cells/well in 48‐well plates for adherent cultures, while incubation with 50 ng mL^−1^ of phorbol myristate acetate (PMA, Absource) for 24 h, followed by additional 48 h in RPMI medium. Afterward, 200 µL of conditioned media of each condition (previously thawed overnight) was added to each well, and incubated for 1 d at 37 °C, in an incubator. As controls, macrophages were also polarized toward a pro‐inflammatory phenotype (M1 phenotype), which occurred following differentiation of macrophages (M0 macrophages) with PMA, followed by an additional 24 h in RPMI medium. On the day after, macrophages were incubated with 20 ng mL^−1^ of recombinant human IFNγ (cat.: 570206, Biolegend) and 100 µg mL^−1^ of LPS (InvivoGen) for 24 h. In the controls having only nonpolarized macrophages (M0) or M1 macrophages, α‐MEM medium frozen at −80 °C and thawed one time was added. Regarding the experiment of IFNγ supplementation, M0 macrophages were established and further incubated for 1 d with α‐MEM medium supplemented with IFNγ at 10 ng mL^−1^. Regarding the experiment of IL‐10 supplementation, M0 macrophages were established and incubated for 1 d with the secretome from the pulsed condition at day 4, with or without the supplementation with 250 pg mL^−1^ of recombinant human IL‐10 (cat.: 571002, BioLegend).

### Endothelial Cell Tube Formation Assay

The conditioned media and Geltrex LDEV‐Free Reduced Growth Factor Basement Membrane Matrix (Thermo Fisher Scientific) were thawed overnight at 4 °C. After, 10 µL of Geltrex were added to each well of a µ‐Slide 15 Well 3D plate (Ibidi), and left solidifying for 1h, 37 °C in an incubator. To keep a moisture environment, the Ibidi plates were placed into 100 mm diameter petri dishes (Sarstedt) with 2 mL DPBS. Briefly, 10 000 HUVECs in 25 µL of M199 supplemented medium were seeded per well of the IbIdi plate. Immediately after, 25 µL of each conditioned media was added in the respective wells, performing a final volume of 50 µL per well, and were left incubating at 37 °C in an incubator for 6 h. As controls, a negative control containing 1:1 of M199 medium to thawed α‐MEM medium was used, as well as a control containing a ratio 1:1 of M199 medium to media of empty alginate beads (“Only alginate”). After 6 h, the morphology of HUVECs was evaluated by Zeiss Primovert microscope, and cells were also stained in the last 30 min with calcein‐AM (ThermoFisher Scientific), at 1:500 in DPBS, 37 °C in an incubator, in the dark. Representative images of each well were taken by widefield fluorescence microscopy (AxioImager2, Zeiss). The images were analyzed using the Angiogenesis Analyzer plugin in ImageJ software.

### Flow Cytometry Analysis

Regarding the experiments with macrophages, nonpolarized and classically polarized macrophages were detached after gentle up and down following incubation with TrypLE Express Enzyme (ThermoFisher Scientific) for 7 min at 37 °C in an incubator, as supplier instructions. Macrophages were centrifuged at 120×*g*, 10 min and incubated for 30 min, RT with FACS buffer—10% (V/V) FBS, 0.1% (W/V) sodium azide (TCI) in PBS–followed by incubation with mouse HLA‐DR/DP/DQ/DX antibody (cat.: SC‐53302, Santa Cruz Biotechnology) at 1:400 in FACS buffer, RT, for 1 h. Afterward, cells were washed once with FACS buffer, 300×*g*, 5 min, RT and incubated with secondary Alexa Fluor 488 goat anti‐mouse antibody (cat.: A‐11029, ThermoFisher Scientific) for 30 min at RT in the dark. After, cells were stained with APC anti‐human CD38 Antibody at 1:100 in FACS buffer (cat.: 303510, BioLegend) for 20 min, RT, in the dark. Cells were then fixed with 1% (V/V) formaldehyde in PBS for 15 min, RT, washed with PBS one time and left in FACS buffer at 4 °C until analyses (up to one week). All samples were acquired on a BD Accuri C6 Plus flow cytometer (BD Biosciences). All data were analyzed using FlowJo v10.7.1 Software.

### Statistical Analysis

Statistical analyses were performed as described in figure legends. All data were analyzed using GraphPad Prism version 9.3.0. For single comparisons, data were analyzed using unpaired *t*‐test, and unless otherwise mentioned, multiple comparisons were analyzed by one‐way ANOVA with Tukey's test. A *p*‐value less than 0.05 was considered statistically significant.

## Conflict of Interest

The authors declare no conflict of interest.

## Author Contributions

A.R.S. and D.C.G. contributed equally to this work. A.R.S., M.B.O., and J.F.M. conceptualized the study and designed the experiments, A.R.S., D.C.G., B.G.N., and A.S.C. conducted experimental work. A.R.S., D.C.G., B.G.N., and A.S.C. analyzed the data. A.R.S. and M.B.O. wrote the manuscript.

## Supporting information

Supporting Information

## Data Availability

The data that support the findings of this study are available from the corresponding author upon reasonable request.
