# Supplementary material for: Encapsulated Mesenchymal Stromal Cells as Cyclic Providers of Immunomodulatory Secretomes: A Living on‐Demand Delivery System
Source: Adv Healthc Mater. 2024 Apr 7;13(17):2304012. doi: 10.1002/adhm.202304012 (PMC11468815; doi:10.1002/adhm.202304012)
Supplement: Supplementary file 1 — Supporting Information [file ADHM-13-2304012-s001.pdf]

ADVANCED HEALTHCARE MATERIALS

Supporting Information

for *Adv. Healthcare Mater.*, DOI 10.1002/adhm.202304012

Encapsulated Mesenchymal Stromal Cells as Cyclic Providers of Immunomodulatory Secretomes: A Living on-Demand Delivery System

Ana Rita Sousa, Diana C. Gonçalves, Beatriz Guapo Neves, Ana Santos-Coquillat, Mariana B. Oliveira and João F. Mano**

Supplementary Information

Encapsulated mesenchymal stromal cells as cyclic providers of immunomodulatory secretomes: a living on-demand delivery system

Ana Rita Sousa[1+], Diana C. Gonçalves[1+], Beatriz G. Neves[1], Ana Santos-Coquillat[1], Mariana B. Oliveira[1*], João F. Mano[1*]

[1]Department of Chemistry, CICECO – Aveiro Institute of Materials, University of Aveiro, Aveiro, Portugal

[+]Equal author contribution

*Corresponding author: Mariana B. Oliveira. E-mail: mboliveira@ua.pt; João F. Mano. E-mail: jmano@ua.pt

Contents

Supplementary Figures

- **Figure S1|** Brightfield images of encapsulated cells while exposed to different regimes of inflammatory stimuli.
- **Figure S2|** Fluorescence images of encapsulated cells while exposed to different regimes of inflammatory stimuli.
- **Figure S3 |** Quantification of de area of living and dead encapsulated cells while exposed to different regimes of inflammatory stimuli.
- **Figure S4|** Effect of different frequencies of IFNγ regimes on secreted factors of mesenchymal stromal cells.
- **Figure S5|** Quantification of IL-10 levels released from a second independent experiment.
- **Figure S6|** Flow cytometry gating strategy for macrophages while incubated with different secretomes.
- **Figure S7|** Flow cytometry gating strategy for macrophages while incubated with different control conditions.
- **Figure S8|** Quantification of pro-inflammatory markers in non-polarized macrophages incubated with IFNγ supplemented medium.
- **Figure S9|** Correlation between the expression of pro-inflammatory markers in macrophages and the IL-10 content of secretomes.
- **Figure S10 |** Flow cytometry gating strategy for macrophages while incubated with IL-10 supplemented secretome.
- **Figure S11 |** Quantification of pro-inflammatory markers in non-polarized macrophages incubated with IL-10 supplemented secretome.
- **Figure S12|** Brightfield images of endothelial cells while exposed to the different regimes of IFNγ.

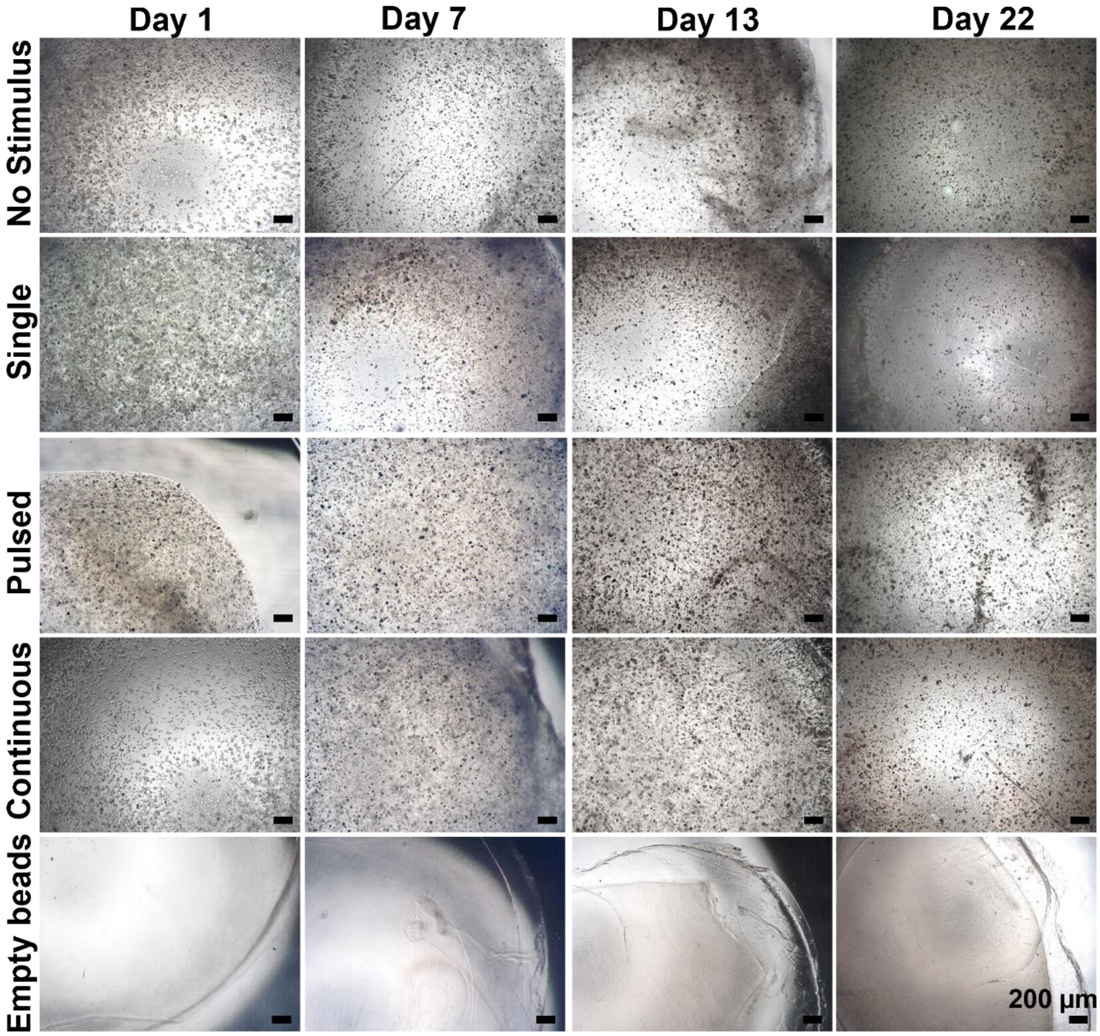

Figure S1 | Brightfield images of encapsulated cells while exposed to different regimes of inflammatory stimuli. Representative brightfield images of human mesenchymal stromal cells derived from the adipose tissue (hASCs) encapsulated in alginate hydrogels after 1, 7, 13 and 22 days. The cells were exposed to different frequencies of IFNγ stimuli – single, pulsed, and continuous. A positive control of encapsulated cells receiving no stimulus was added, as a negative control of empty beads. Scale bar: 200 µm.

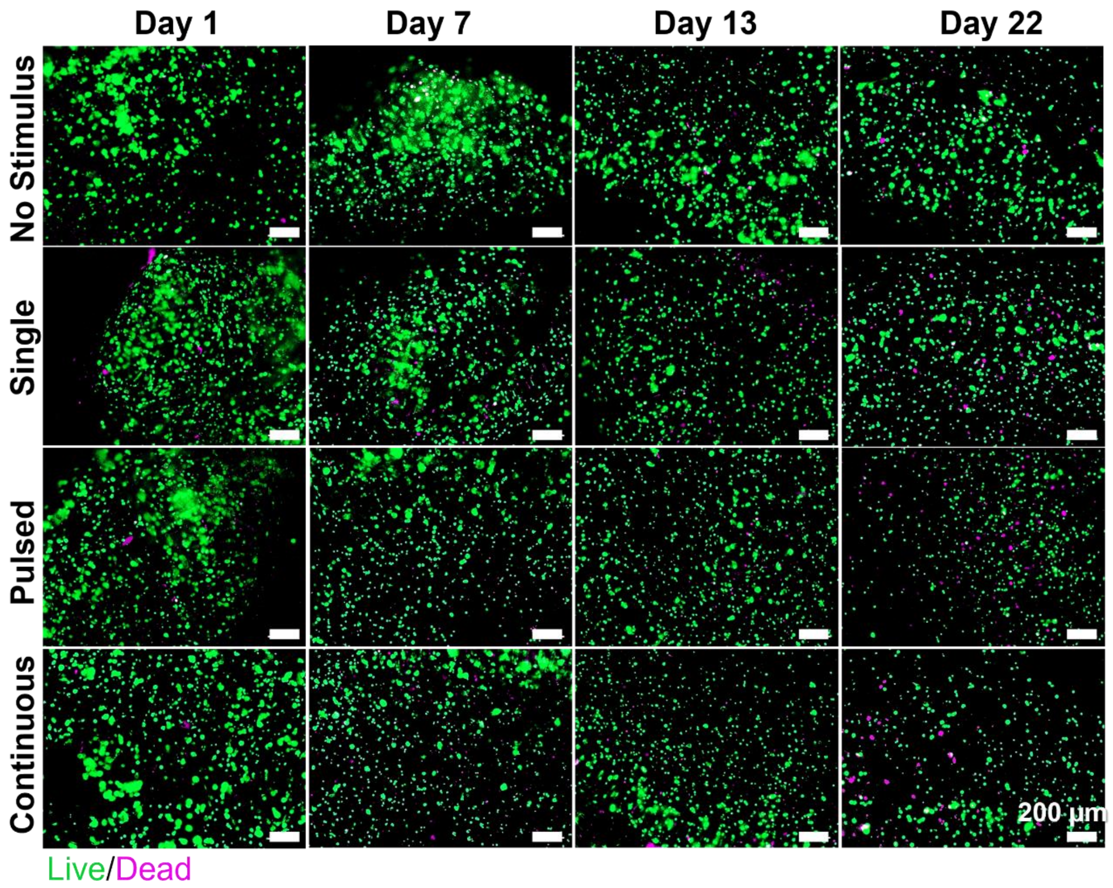

Figure S2 | Fluorescence images of encapsulated cells while exposed to different regimes of inflammatory stimuli. Representative fluorescence images of human mesenchymal stromal cells derived from the adipose tissue (hASCs) encapsulated in alginate hydrogels after 1, 7, 13 and 22 days. Encapsulated hASCs were stained for living (calcein, green) and dead (PI, magenta) cells. The cells were exposed to different frequencies of IFNγ stimuli – single, pulsed, and continuous. A positive control of encapsulated cells receiving no stimulus was added. Scale bar: 200 µm.

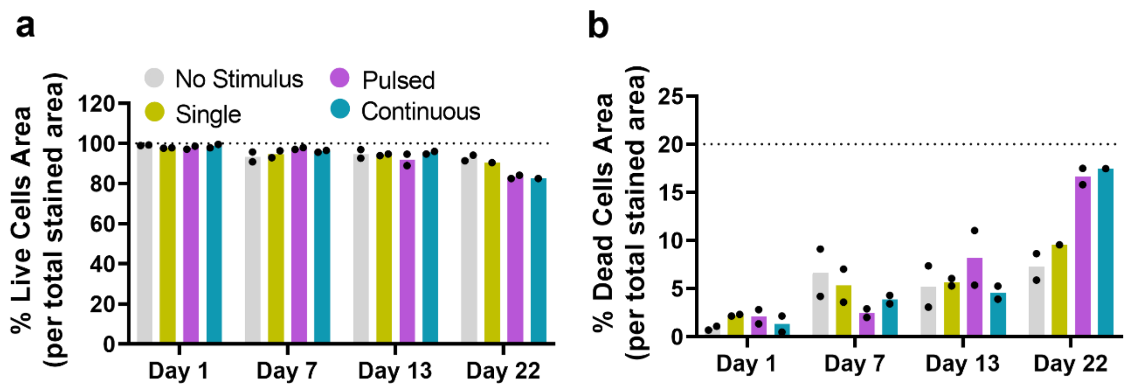

Figure S3 | Quantification of de area of living and dead encapsulated cells while exposed to different regimes of inflammatory stimuli. Quantification of the area of living and dead cells was performed from representative fluorescence images of human mesenchymal stromal cells derived from the adipose tissue (hASCs) encapsulated in alginate hydrogels after 1, 7, 13 and 22 days. Encapsulated hASCs were stained for living (calcein) and dead (PI) cells. All data was normalized to the total stained area.

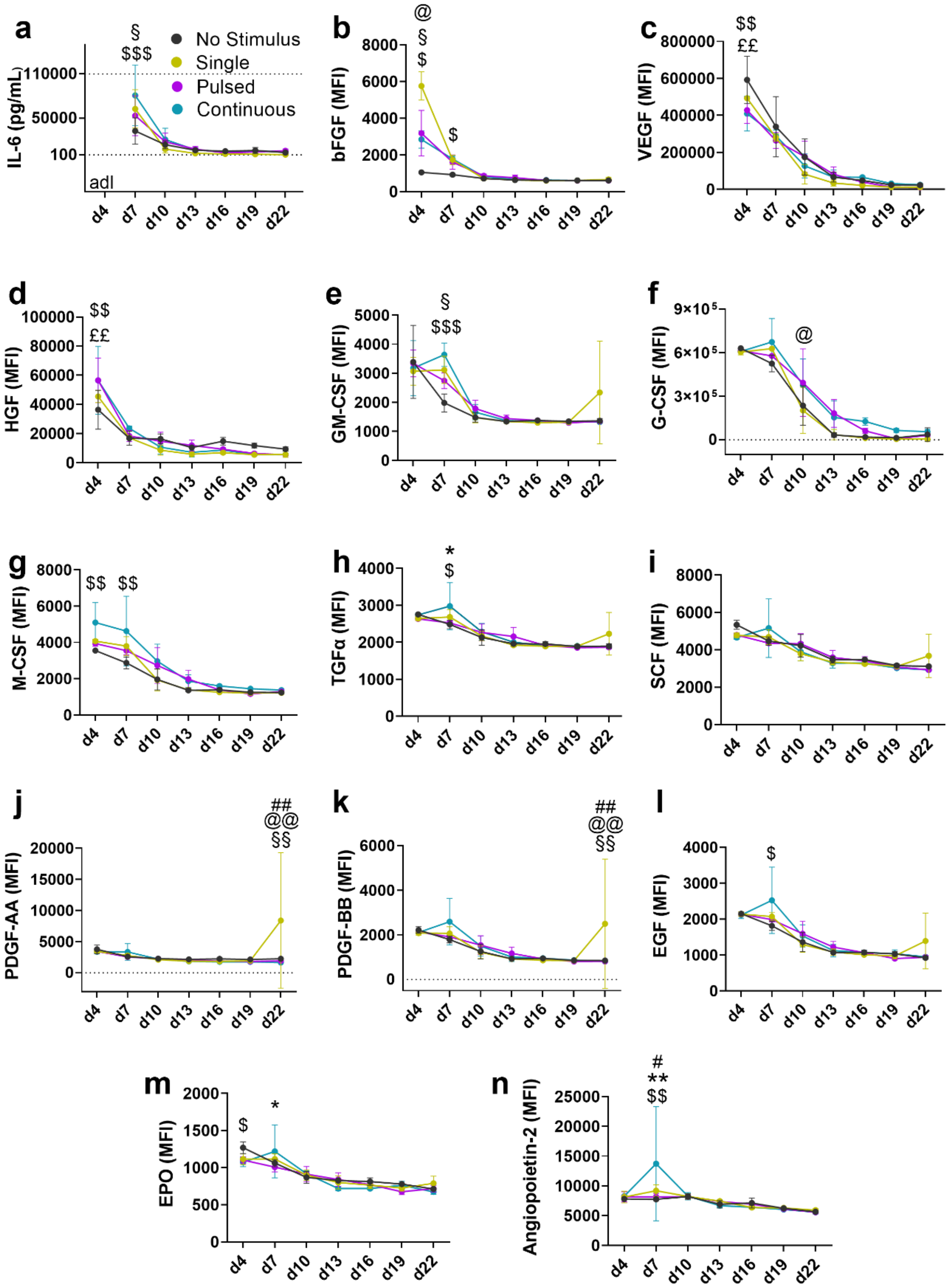
a
b
c
d
e
f
g
h
i
j
k
l
m
n
No Stimulus
Single
Pulsed
Continuous
IL-6 (pg/mL)
bFGF (MFI)
VEGF (MFI)
HGF (MFI)
GM-CSF (MFI)
G-CSF (MFI)
M-CSF (MFI)
TGFα (MFI)
SCF (MFI)
PDGF-AA (MFI)
PDGF-BB (MFI)
EGF (MFI)
EPO (MFI)
Angiopoietin-2 (MFI)
adl
d4
d7
d10
d13
d16
d19
d22

Figure S4 | Effect of different frequencies of IFNγ regimes on secreted factors of mesenchymal stromal cells. Quantification of pro-regenerative and immunomodulatory factors released from human mesenchymal stromal cells from the adipose tissue (hASCs) encapsulated into alginate hydrogels and submitted to different frequencies of IFNγ stimuli – single, pulsed and continuous –, over 22 days. **a** Quantification of the secretion of IL-6 was performed through ELISA assay. The dashed line on top depicts the above detection limit (adl) and the lower dashed line represents the below detection limit (bdl). The remaining molecules were quantified by a multiplex of 13 molecules through a LEGENDplex™ kit. **b-c** Quantification of molecules of pro-angiogenic potential. **d** Quantification of molecules of pro-regenerative and trophic potential; **e-f** Quantification of molecules of immunosuppressive function; **g-i** Quantification of immunomodulatory molecules; as well as other growth factors and pro-regenerative molecules **j-n**. The symbols '#' refer to significative differences between single and continuous conditions; '*': refer to differences between pulsed and continuous stimulus; '$': refer to positive control and continuous stimulus; '@': refer to differences between single and pulsed regimens; '£': refer to differences between positive control and pulsed regimens; '§': refer to differences between positive control and single stimulus. n=3 biological replicates. Error bars are mean ± SD. Statistical differences were determined using Two-way ANOVA followed by Tukey's multiple comparisons test.

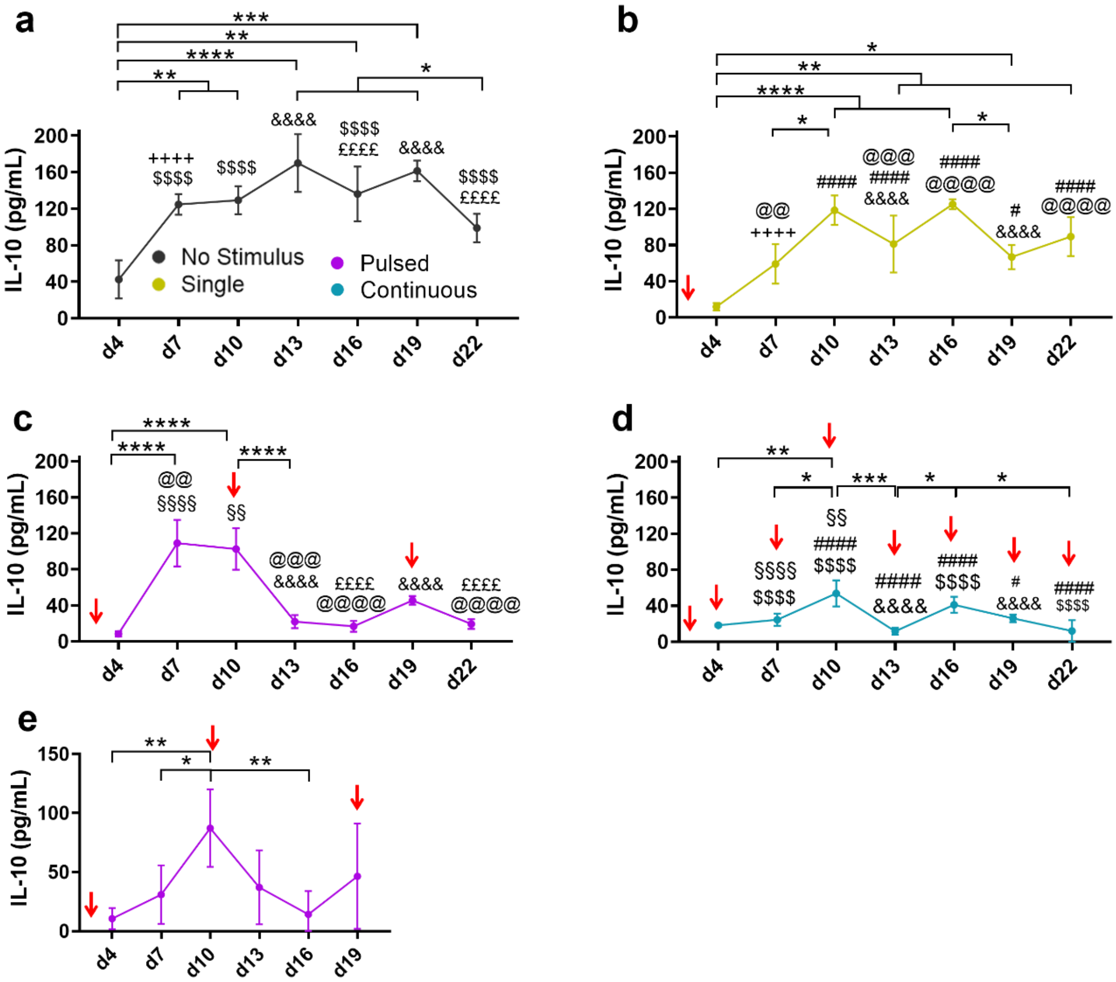

Figure S5 | Quantification of IL-10 levels released from a second independent experiment. a Quantification of secreted IL-10 molecules from a second independent experiment by ELISA assay, according to the different IFNγ frequencies – no stimulus, single **b**, pulsed **c**, and continuous **d** –, over 22 days. **e** Quantification of secreted IL-10 from a third independent experiment using a different hASCs donor, under the pulsed stimulation regime. To determine differences over time within each condition One-way ANOVA followed by Tukey's multiple comparisons test was used, represented by '*' symbol. To determine differences between different frequencies of inflammatory stimuli, Two-way ANOVA followed by Tukey's multiple comparisons test was used. The symbols '+' refer to significative differences between no stimulus and single stimulus; '§': differences between pulsed and continuous stimulus; '&': differences between no stimulus and all remaining conditions (single, pulsed, continuous); '$': differences between no stimulus and continuous stimulus; '#': differences between single and continuous conditions; '@': differences between single and pulsed conditions; '£':

differences between no stimulus and pulsed stimulus. Red arrows indicate the timing of IFNγ application. n=3 biological replicates. Error bars are mean ± SD.

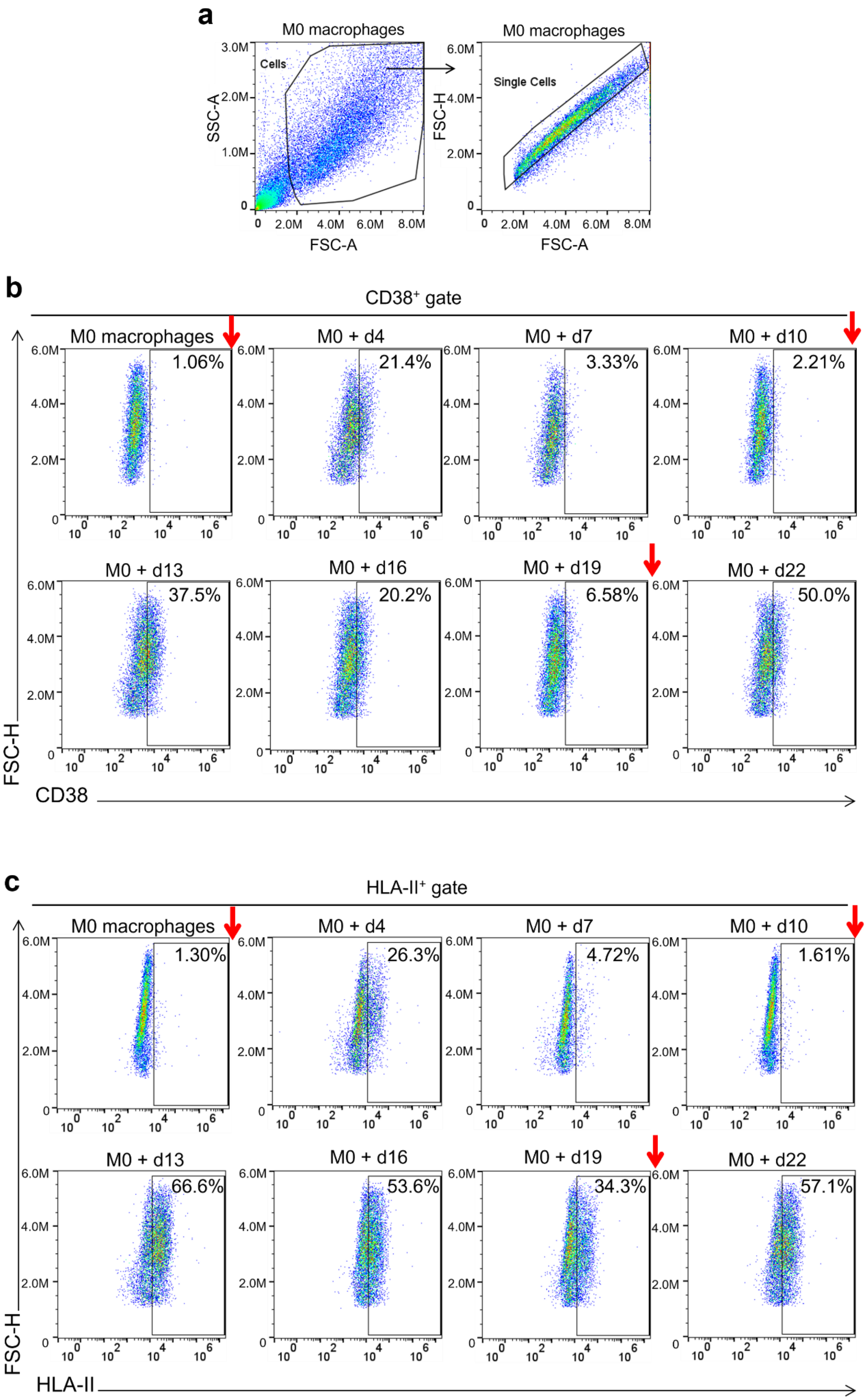
a
M0 macrophages
Cells
SSC-A
FSC-A
M0 macrophages
Single Cells
FSC-H
FSC-A
b
CD38⁺ gate
M0 macrophages
1.06%
M0 + d4
21.4%
M0 + d7
3.33%
M0 + d10
2.21%
M0 + d13
37.5%
M0 + d16
20.2%
M0 + d19
6.58%
M0 + d22
50.0%
FSC-H
CD38
c
HLA-II⁺ gate
M0 macrophages
1.30%
M0 + d4
26.3%
M0 + d7
4.72%
M0 + d10
1.61%
M0 + d13
66.6%
M0 + d16
53.6%
M0 + d19
34.3%
M0 + d22
57.1%
FSC-H
HLA-II

Figure S6 | Flow cytometry gating strategy for macrophages while incubated with different secretomes. Representative gating strategy applied in all conditions of non-polarized macrophages derived from THP-1 cell line (M0 macrophages) **a**, while incubated for one day with secretomes collected from the pulsatile condition, showcasing the pro-inflammatory CD38 **b**, and HLA-II markers, **c**. All secretomes used were freeze-thawed one time before application. Red arrows indicate the timing of IFNγ application in encapsulated mesenchymal stromal cells.

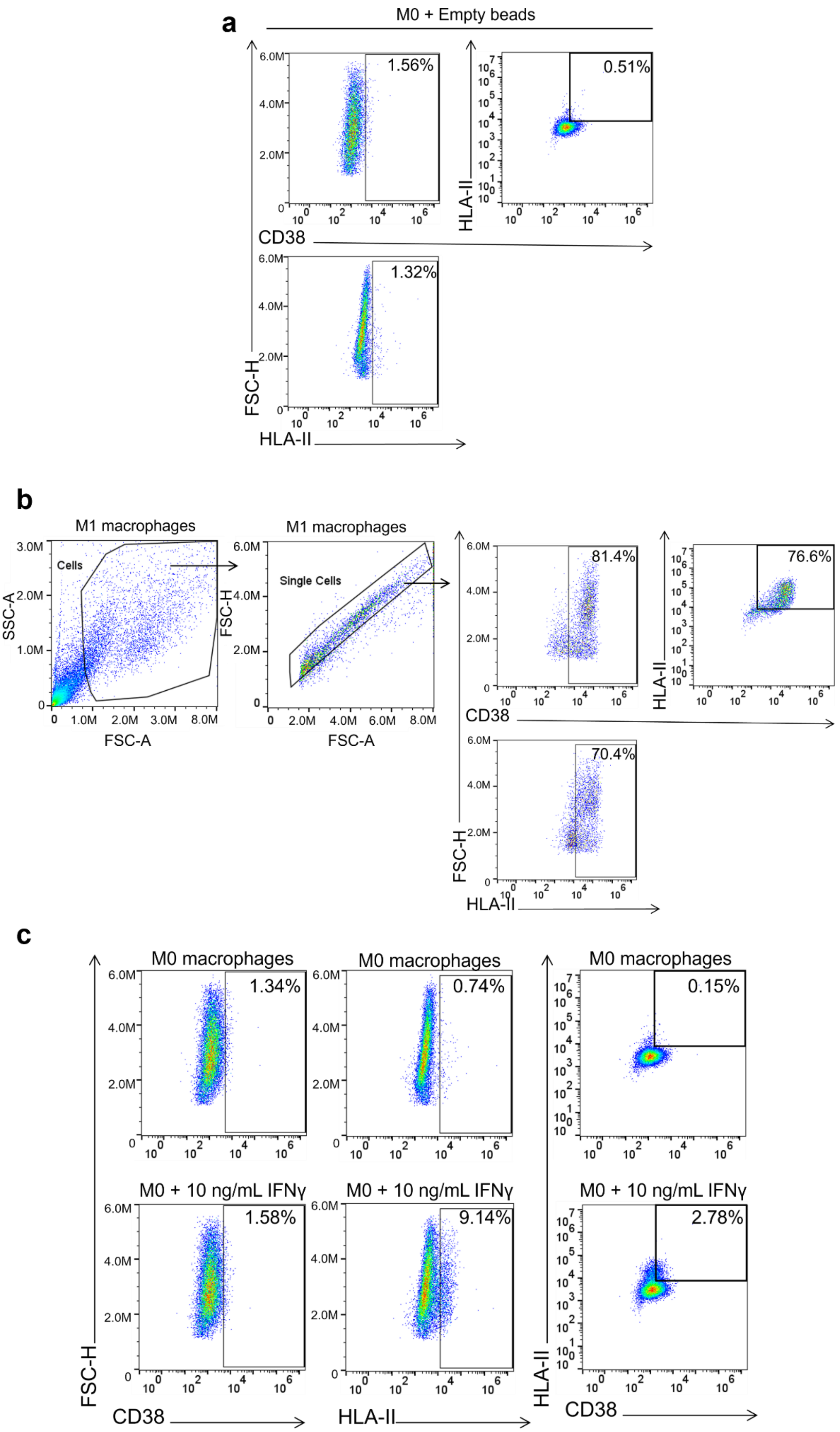
a
M0 + Empty beads
1.56%
0.51%
1.32%
CD38
HLA-II
FSC-H
b
M1 macrophages
Cells
Single Cells
SSC-A
FSC-A
FSC-H
81.4%
76.6%
70.4%
CD38
HLA-II
c
M0 macrophages
M0 + 10 ng/mL IFNγ
1.34%
0.74%
0.15%
1.58%
9.14%
2.78%
FSC-H
CD38
HLA-II

Figure S7 | Flow cytometry gating strategy for macrophages while incubated with different control conditions. Representative gating strategy for CD38 and HLA-II markers expressed by macrophages derived from the THP-1 cell line following 1 day incubation with different controls. **a**, Non-polarized macrophages were incubated with the leachables of empty beads condition. **b**, A control of classically polarized (M1) macrophages was incubated with cell culture medium. **c**, Non-polarized macrophages were incubated with a control of 10 ng/mL of IFNγ. All leachables or cell culture medium used were freeze-thawed one time before application.

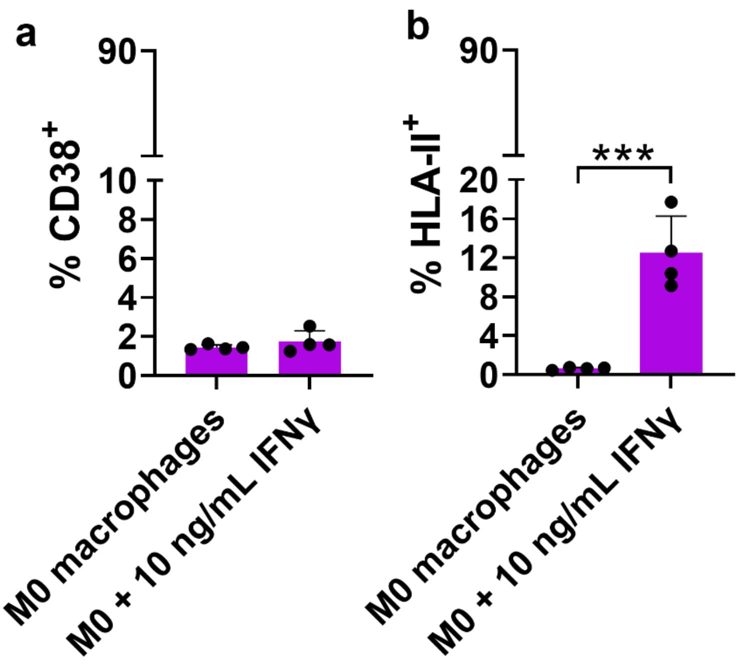

FigureS8 | Quantification of pro-inflammatory markers in non-polarized macrophages incubated with IFNγ supplemented medium. Quantification of the percentage of $CD38^+$ cells, **a**, or $HLA\text{-}II^+$ cells, **b**, while non-polarized macrophages were incubated for one day with cell culture medium or 10 ng/mL IFNγ supplemented medium. The cell culture medium or IFNγ-supplemented medium used were freeze-thawed one time before application. Statistical analyses were performed using Unpaired t test. n = 4 biological replicates, error bars are mean ± SD.

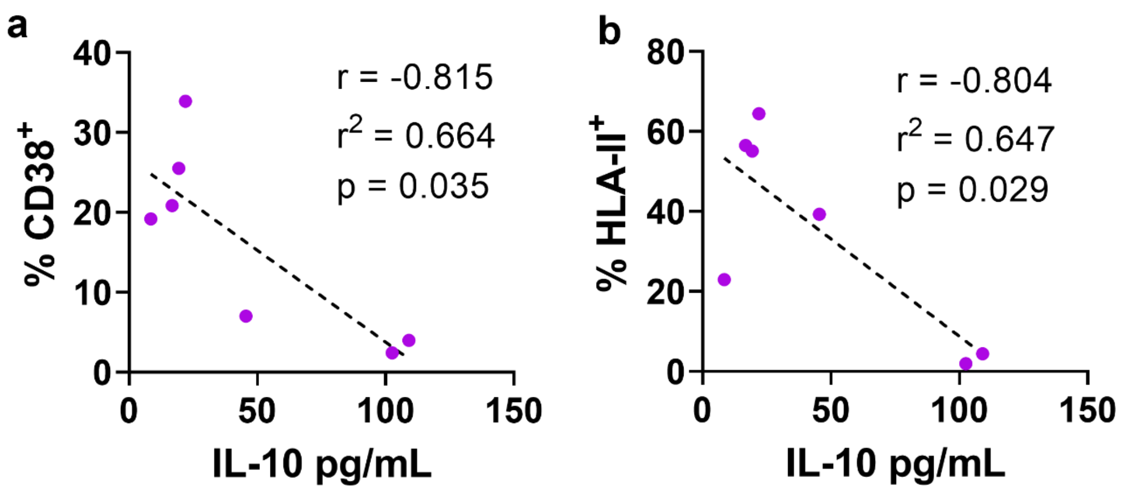

Figure S9 | Correlation between the expression of pro-inflammatory markers in macrophages and the IL-10 content of secretomes. Correlation between the percentage of $CD38^+$ cells **a**, or HLA-II^+ cells **b**, in a population of THP-1 derived non-polarized macrophages while incubated with the secretomes collected at different days from the pulsed condition, with the IL-10 concentration of those secretomes, using the parametric Pearson test.

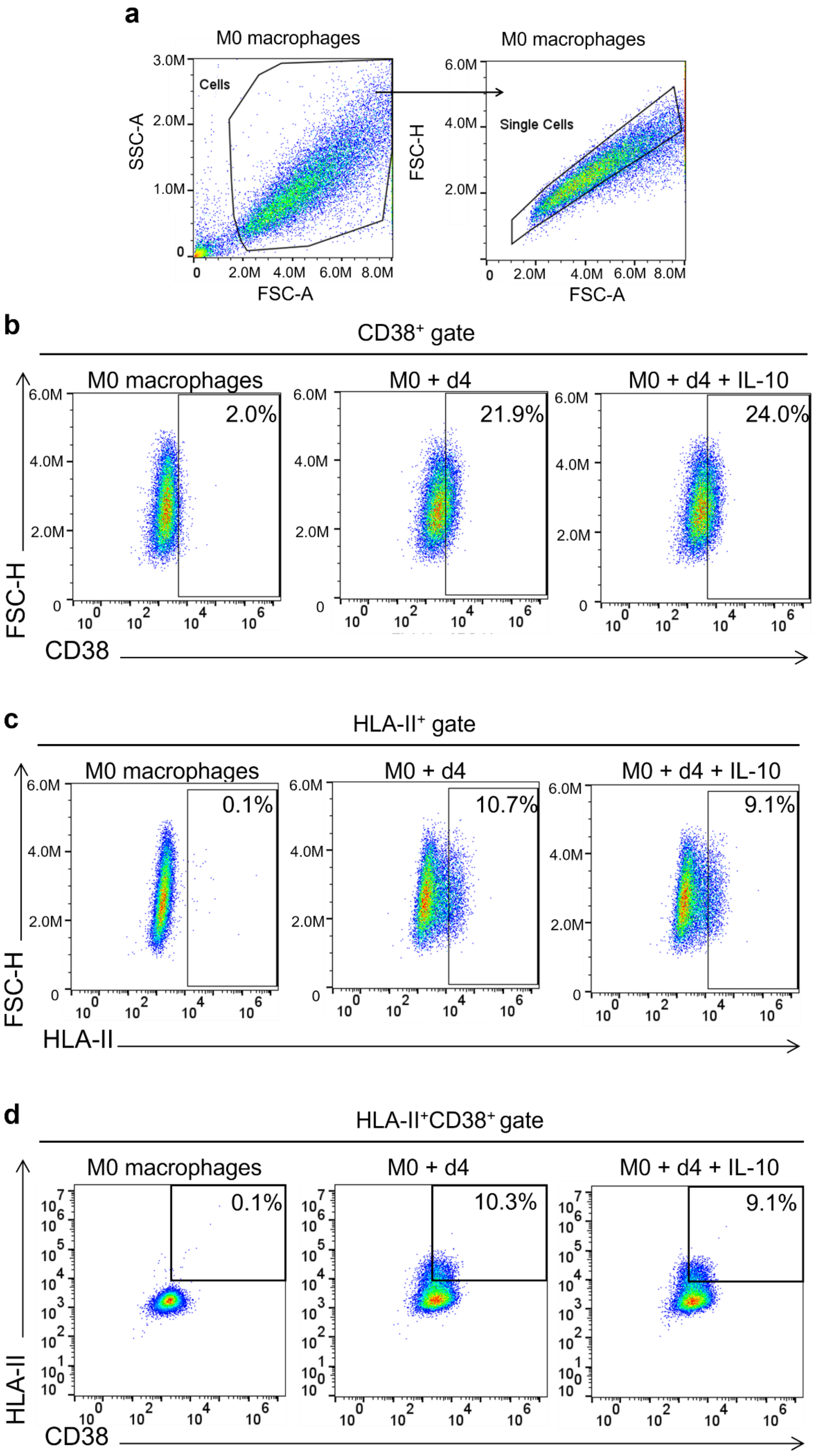

a
M0 macrophages
Cells
SSC-A
FSC-A
Single Cells
FSC-H
b
CD38+ gate
M0 macrophages
M0 + d4
M0 + d4 + IL-10
2.0%
21.9%
24.0%
FSC-H
CD38
c
HLA-II+ gate
M0 macrophages
M0 + d4
M0 + d4 + IL-10
0.1%
10.7%
9.1%
FSC-H
HLA-II
d
HLA-II+CD38+ gate
M0 macrophages
M0 + d4
M0 + d4 + IL-10
0.1%
10.3%
9.1%
HLA-II
CD38

Figure S10 | Flow cytometry gating strategy for macrophages while incubated with IL-10 supplemented secretome. Representative gating strategy applied in all conditions of non-polarized macrophages derived from THP-1 cell line (M0 macrophages) **a**, while incubated for one day with secretome collected from the pulsatile condition at day 4 supplemented or not with 250 pg/mL of IL-10, showcasing the pro-inflammatory CD38 **b**, and HLA-II markers **c**, All secretomes used were freeze-thawed one time before application. As control, M0 macrophages were incubated with α-MEM cell culture medium.

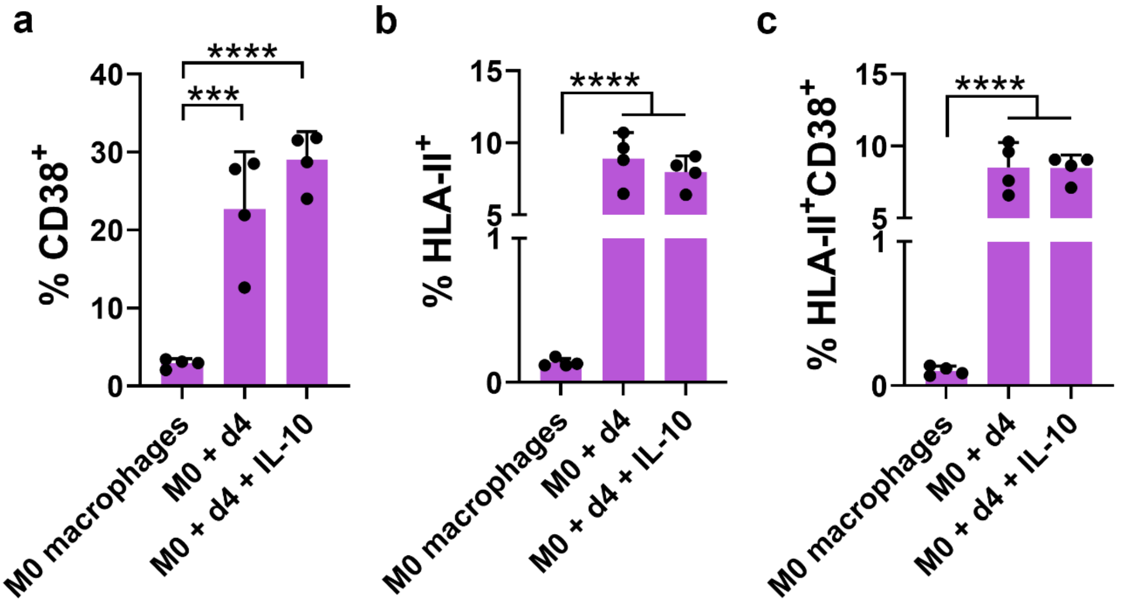

Figure S11 | Quantification of pro-inflammatory markers in non-polarized macrophages incubated with IL-10 supplemented secretome. Quantification of the percentage of $CD38^+$ cells **a**, $HLA\text{-}II^+$ cells **b**, or $HLA\text{-}II^+CD38^+$ cells **c**, while non-polarized macrophages were incubated for one day with secretome collected from the pulsatile condition at day 4 supplemented or not with 250 pg/mL of IL-10. As control, M0 macrophages were incubated with α-MEM cell culture medium. All secretomes used were freeze-thawed one time before application. Statistical analyses were performed using One-way ANOVA followed by Tukey's multiple comparisons. n = 4 biological replicates, error bars are mean ± SD.

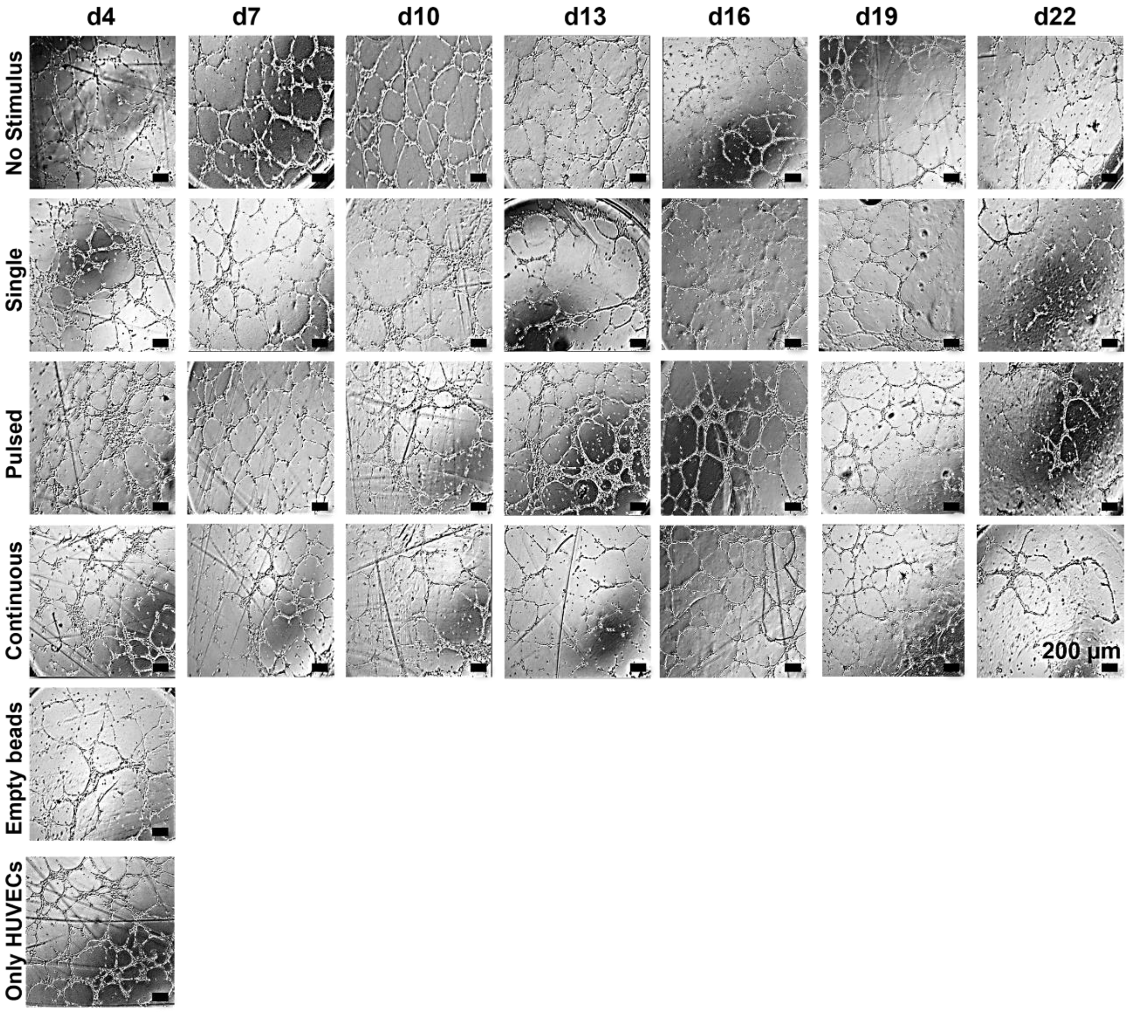

Figure S12 | Brightfield images of endothelial cells while exposed to the different regimes of IFNγ. Representative brightfield images of human umbilical vein endothelial cells (HUVECs) while incubated for 6 h with a ratio of 1:1 of M199 medium to secretomes collected from different IFNγ regimes over time – single, pulsed, and continuous. As controls, HUVECs were either incubated with secretome collected from encapsulated cells receiving no stimulus, or, as negative controls, HUVECs were incubated with the leachables of alginate beads (Empty beads) or α-MEM medium (Only HUVECs). Scale bar: 200 µm.
